# Microbial epibiotic community of the deep-sea galatheid squat lobster *Munidopsis alvisca*

**DOI:** 10.1038/s41598-022-06666-x

**Published:** 2022-02-17

**Authors:** Janina Leinberger, Felix Milke, Magdalini Christodoulou, Anja Poehlein, Javier Caraveo-Patiño, Andreas Teske, Thorsten Brinkhoff

**Affiliations:** 1 Institute for Chemistry and Biology of the Marine Environment, University of Oldenburg, Oldenburg, Germany; 2 German Centre for Marine Biodiversity Research (DZMB), Senckenberg am Meer, Wilhelmshaven, Germany; 3 Genomic and Applied Microbiology and Göttingen Genomics Laboratory, Institute of Microbiology and Genetics, University of Göttingen, Göttingen, Germany; 4 Centro de Investigaciones Biológicas del Noroeste (CIBNOR), La Paz, Mexico; 5 Department of Earth, Marine and Environmental Sciences, University of North Carolina at Chapel Hill, Chapel Hill, USA

**Keywords:** Microbiology, Zoology

## Abstract

Life at hydrothermal vent sites is based on chemosynthetic primary producers that supply heterotrophic microorganisms with substrates and generate biomass for higher trophic levels. Often, chemoautotrophs associate with the hydrothermal vent megafauna. To investigate attached bacterial and archaeal communities on deep-sea squat lobsters, we collected ten specimens from a hydrothermal vent in the Guaymas Basin (Gulf of California). All animals were identified as *Munidopsis alvisca* via morphological and molecular classification, and intraspecific divergence was determined. Amplicon sequencing of microbial DNA and cDNA revealed significant differences between microbial communities on the carapaces of *M. alvisca* and those in ambient sea water. Major epibiotic bacterial taxa were chemoautotrophic *Gammaproteobacteria*, such as *Thiotrichaceae* and *Methylococcaceae*, while archaea were almost exclusively represented by sequences affiliated with *Ca. Nitrosopumilus*. In sea water samples, Marine Group II and III archaea and organoheterotrophic *Alphaproteobacteria*, *Flavobacteriia* and *Planctomycetacia* were more dominant. Based on the identified taxa, we assume that main metabolic processes, carried out by *M. alvisca* epibiota, include ammonia, methane and sulphide oxidation. Considering that *M. alvisca* could benefit from sulphide detoxification by its epibiota, and that attached microbes are supplied with a stable habitat in proximity to substrate-rich hydrothermal fluids, a mutualistic host-microbe relationship appears likely.

## Introduction

Deep-sea hydrothermal vent sites are extreme marine habitats, often characterised by low oxygen levels and high concentrations of reduced chemical compounds that originate from emissions of hydrothermal fluids or pyrolysis of organic matter^1,2^. Life at those sites is generally assumed to be sustained by chemosynthetic primary producers and their interactions with other organisms^3^. By oxidation of reduced species, such as ammonia or hydrogen sulphide, chemoautotrophic microbes generate biomass and provide substrates for organoheterotrophs^1,4–6^. In addition, chemosynthetic microbes are often found in association with the hydrothermal vent macro- and megafauna, for instance decapod crustaceans like *Austinograea* sp.^3^, *Rimicaris exoculata*^7^, *Shinkaia crosnieri*^8^ or *Kiwa hirsuta*^9^.

Two major aspects under debate are the ecological relevance of these epibiotic bacteria for their host and whether microbial symbionts are obtained from conspecific animals or the environment^10^. A major drawback to answering these questions is the low number of sites and organisms that have been sampled^11^. Observations of the deep-sea shrimp *R. exoculata* and the squat lobster *S. crosnieri* indicate an at least partially nutritional role of symbionts for the crustaceans. It is assumed that *R. exoculata* “farm” their symbionts and take up microbial metabolic products^7^. To continuously supply their symbionts with reduced components from hydrothermal vent fluids, the shrimps gather around active chimneys^12^.

An extensively studied example for a hydrothermally active deep-sea environment is the Guaymas Basin in the Gulf of California. Due to high biological productivity in the overlying waters, this young, active seafloor-spreading centre has exceptionally fast sedimentation rates^13^. Microbial mats and hydrothermal sediments in the Guaymas Basin are rich in CO_2_, H_2_, low molecular-weight organic acids, ammonia, methane and light hydrocarbons that serve as substrates for microbial oxidative processes^2^. Common chemoautotrophs found in sediments and waters throughout the Guaymas Basin include members of the *Gamma*- and *Epsilonproteobacteria* as well as *Thaumarchaeota*, of which the latter are usually strongly represented by the genus *Ca. Nitrosopumilus*^14,15^. While these bacterial taxa were also frequently found in association with deep-sea hydrothermal vent crustaceans^12,16^, little is known about archaeal epibionts of the deep-sea megafauna. In proximity to the Guaymas Basin vents, decapod crustaceans of the genus *Munidopsis* are commonly found^17^. *Munidopsis* is the second largest genus of squat lobsters, and its members have a broad bathymetric and geographic distribution^11,18^. However, there are no studies describing the composition and diversity of the epibiota, or investigating the potential role of the epibionts in host ecology, even though *Munidopsis* spp. are considered as “classical” and ubiquitous deep-sea fauna^19–21^.

The purpose of the present study was to describe the composition and activity of epibiotic microbial communities, living on the carapace of *Munidopsis alvisca*, under ecological aspects*.* For species delimitation, we aimed to determine intra- and inter-species genetic divergence for *M. alvisca*. We collected *M. alvisca* specimens at Rebecca’s Roost, one of the largest hydrothermal spires in the Guaymas Basin^13^, at a depth of 2000 m. Amplicon sequencing was applied to receive a high taxonomic resolution of the epibiotic microbial community. Special attention was paid to distinct differences of the bacterial and archaeal community found on male and female squat lobsters, as well as in the surrounding sea water. Finally, our data was correlated with existing database entries and literature reports to infer potential host-microbe and microbe-microbe interactions.

## Results

### Sampling site description

Decapods were collected on rocky terrain at the base of a major hydrothermal structure (Rebecca’s Roost) where core sampling and temperature probe insertion was not possible. The animals were immersed in cold Guaymas Basin bottom water, at a consistent in situ temperature of 3 °C and an oxygen concentration of 10–15% saturation. These conditions persist very closely to hydrothermally active sites, and change to hot and reducing only after crossing the interface, for example within surficial sediment^13,22^. The mosaic of rust-coloured and sulphur-encrusted rock surfaces at the base of Rebecca’s Roost indicated a patchwork of generally oxidising conditions with local reducing spots between which the animals can move to find their preferred location, biogeochemical regime and food source. While sulphide and other hydrothermal gases that seep from hot sediments around Rebecca’s Roost quickly mix with sea water and do not accumulate, the mobile crabs have easy access to local areas with hydrothermal flow, warm seepage and chemosynthetically generated biomass. Good examples are the hot, sulphide- and methane-rich sediment cores 4870–16 and 4870–29 collected at Cathedral Hill ca. 150 m from Rebecca’s Roost^23,24^. Further, local bottom waters are rich in nitrogen species i.e. ammonia, nitrate and nitrite as described in^25^.

### Integrative taxonomic identification of *Munidopsis alvisca* Williams, 1988

**Figure 1:**
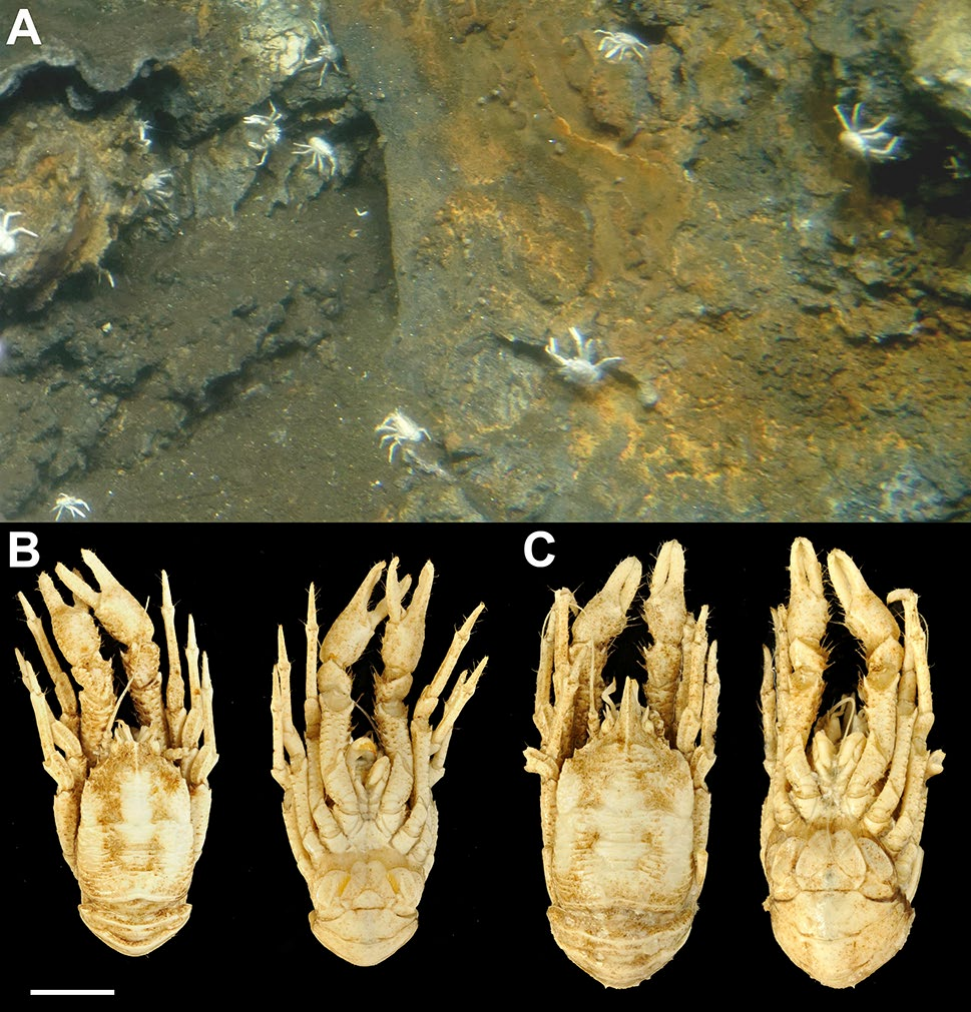
*Munidopsis alvisca* specimens (**A**) at the hydrothermal vent site Rebecca’s Roost (Guaymas Basin, Mexico) at 1996 m depth (*Alvin* dive 4995). Animals were collected with the submersible’s slurp gun. Dorsal and ventral views of (**B**) a male and (**C**) a female *M. alvisca* specimen (scale 1 cm) collected at Rebecca’s Roost. Pictures taken by Thorsten Brinkhoff and Nicol Mahnken.

**Figure 2:**
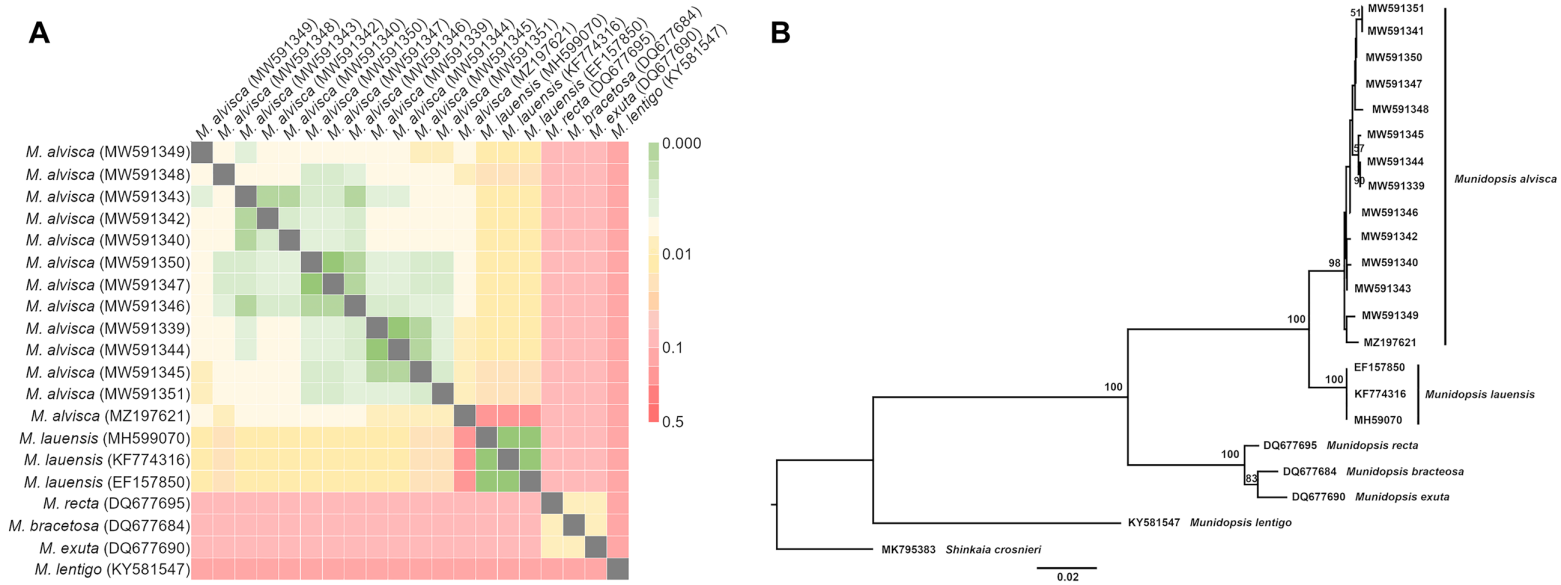
(**A**) Mean nucleotide distances (p-distance) of cytochrome c oxidase I (COI) gene sequences within *Munidopsis alvisca* specimens (this study and MZ197621) and in comparison with other *Munidopsis* hydrothermal vent species, indicated by color code. (**B**) Neighbor-joining tree (p-distance) based on *Munidopsis alvisca* COI DNA barcodes. Bootstrap values ≥ 50% (derived from 1000 replicates) at main nodes are shown. References for sequences and values for p-distances are given in Supplement T1.

We identified all ten collected squat lobsters as representatives of the species *Munidopsis alvisca* Williams, 1988 based on the following morphological characteristics: absence of pairs of epigastric spines on the carapace, a smooth carapace with transverse rugae, rostrum without pairs of lateral spines, absence of epipods on P1-P4, cornea not depressed, long eyespine strongly produced, posteromedian margin of the sixth pleomere transverse, dactyli terminal claw of P2-P4 strongly curving^20^. The *M. alvisca* specimens collected at Rebecca’s Roost agree well with the original description and occur locally in high densities (Fig. 1A). None of the animals showed any lesions or other morphological alterations of the carapace that would indicate shell disease (Fig. 1B, C). Out of the ten specimens selected for analysis of epibiotic microbial communities, five were identified as males and five as females. The intraspecific sequence divergence (p-distance), calculated by comparison of mitochondrial cytochrome c oxidase subunit I (COI) gene sequences of collected *M. alvisca* specimens, ranged between 0.15–1.07% (Fig. 2, Supplement T1). The mean interspecific sequence divergence, when compared with other *Munidopsis* vent species, ranged between 2.4% (with *Munidopsis lauensis*) and 25.0% (*Munidopsis lentigo*) (Fig. 2, Supplement T1).

### Sequencing data of the attached microbial community on *M. alvisca* specimens

Using amplicon sequencing of bacterial and archaeal 16S rRNA genes and their transcripts, five male and five female *M. alvisca* specimens were investigated for their epibiotic microbial community and compared to the free-living community in the ambient sea water. After bioinformatic quality control, a total of 237,723 and 211,909 reads were obtained for bacterial DNA and cDNA, respectively, and 225,323 and 77,820 reads for archaeal DNA and cDNA, respectively. Transcribed RNA (cDNA) was sequenced and analysed as a measure for microbial activity. Amplicon sequence variants (ASVs) with ≥ 5 reads were considered for further analysis, yielding 2037 and 1965 bASVs for bacterial DNA and cDNA, and 364 and 111 aASVs for archaeal DNA and cDNA, respectively.

### Diversity of free-living and epibiotic microbial communities of *Munidopsis alvisca* and differences between sample types

**Figure 3:**
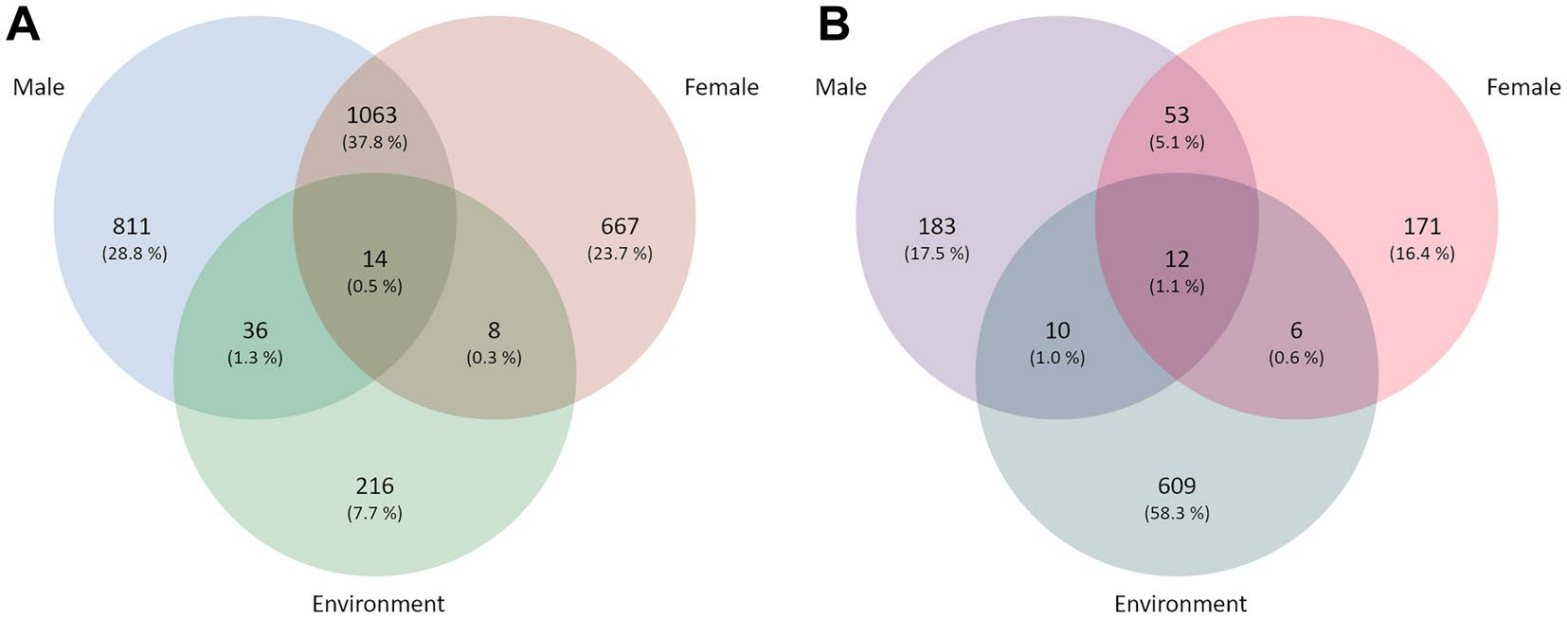
Venn-diagrams showing ASVs in (**A**) the archaeal and (**B**) the bacterial datasets that are unique to or shared between different sample types (male/female/environment). Numbers refer to total ASVs found in the combined DNA and cDNA datasets. The share of the sum of ASVs is given as percentage in brackets behind each number.

Our dataset features several bacterial and archaeal ASVs that are highly abundant in the cDNA dataset, but are not detected in the DNA dataset. To avoid underrepresentation of rare taxa with high 16S rRNA transcript numbers, in the comparison of different sample types (female/male/environment) total ASVs (combined DNA and cDNA datasets) are shown and referred to. Both, bacterial and archaeal ASVs showed very low overlap between epibiota and sea water (Fig. 3). Most ASVs were exclusive to either sample type (male, female, sea water) except epibiotic bASVs, of which 38% were found on both sexes. The most pronounced difference between the bacterial and archaeal dataset was that the majority of bASVs (90.3%) was exclusively found on squat lobsters (Fig. 3A), while the majority of aASVs (58.3%) was only present in the sea water samples (Fig. 3B).

**Figure 4:**
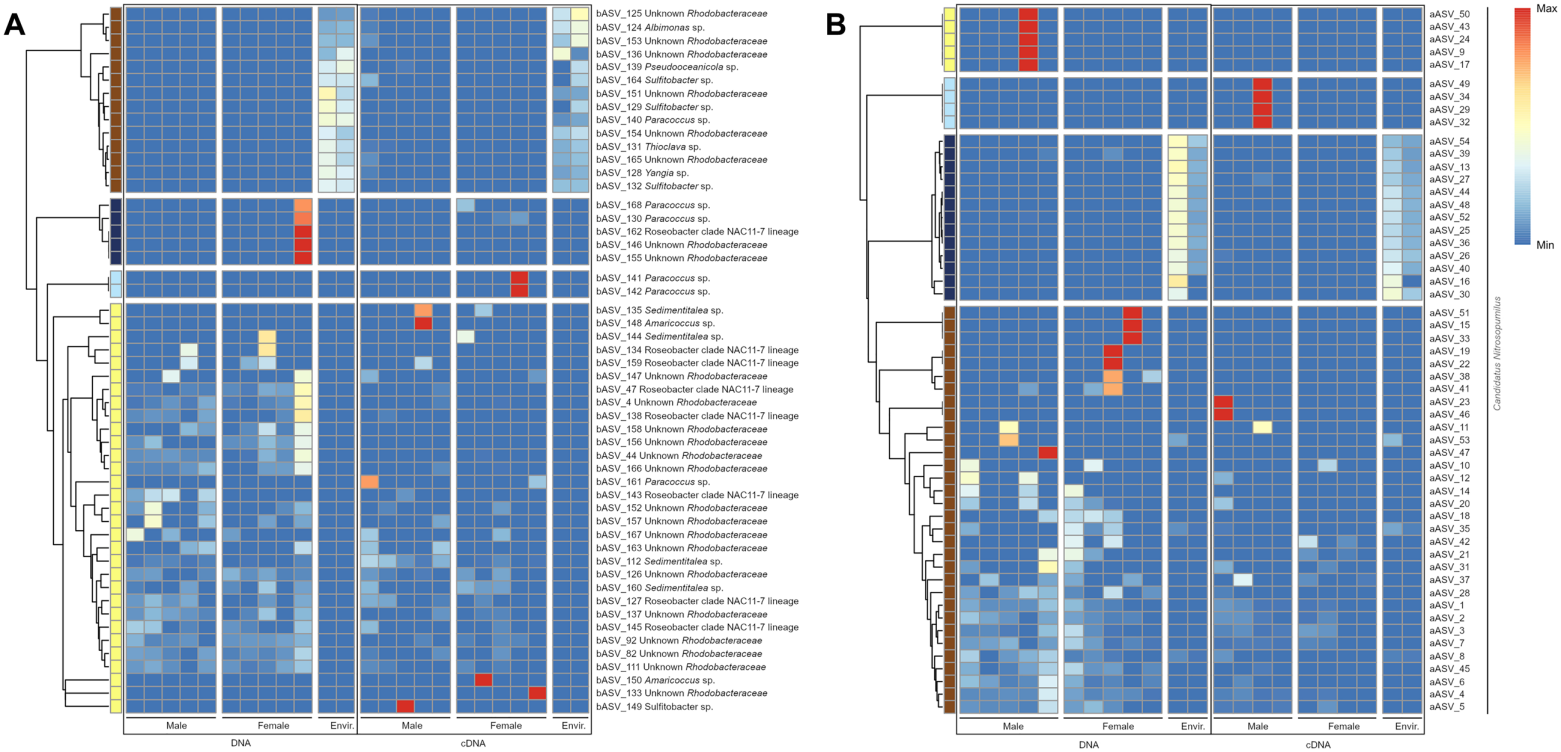
Relative abundances of individual ASVs within selected (**A**) bacterial and (**B**) archaeal taxa, shown exemplarily for (**A**) *Rhodobacteraceae* and (**B**) *Ca. Nitrosopumilus* to highlight microdiversity on lower taxonomic ranks. In (B), no ASVs in the cDNA dataset from two *M. alvisca* specimens (one female, one male) matched the selection criteria defined in the methods section. Therefore, only four females and four males are included in the figure. Compositional relatedness based on Bray–Curtis distances is displayed left of each heatmap. max/min—relative maximum/minimum abundance within the displayed taxon. Identifiers for each ASV are listed in Supplement T3,T4.

Further, each sample was characterised by a variety of distinct oligotypes of the same taxonomic unit, thus showing a unique “fingerprint” of detected sequences, some of which were highly active (Fig. 4). This microdiversity was extremely pronounced in several bacterial families like *Rhodobacteraceae* or *Methylomonaceae* and in the archaeal dataset, in which all samples were dominated by the genus *Nitrosopumilus* (Fig. 4).

#### Comparison of microbial communities: free-living versus attached communities

Microbial communities on *M. alvisca* carapaces were significantly different from those in the free-living fraction. This was found in both, the RNA (p_Archaea_ = 0.001 and p_Bacteria_ = 0.007) and the DNA dataset (p_Archaea_ = 0.006 and p_Bacteria_ = 0.006). Additionally, in all samples the archaeal diversity was distinctly lower than the bacterial diversity. While the bacterial epibiotic diversity was higher than that of sea water samples, the opposite was found for the archaeal communities (Supplement T2).

#### Comparison of microbial communities: assemblages on male and female *M. alvisca* specimens

**Figure 5:**
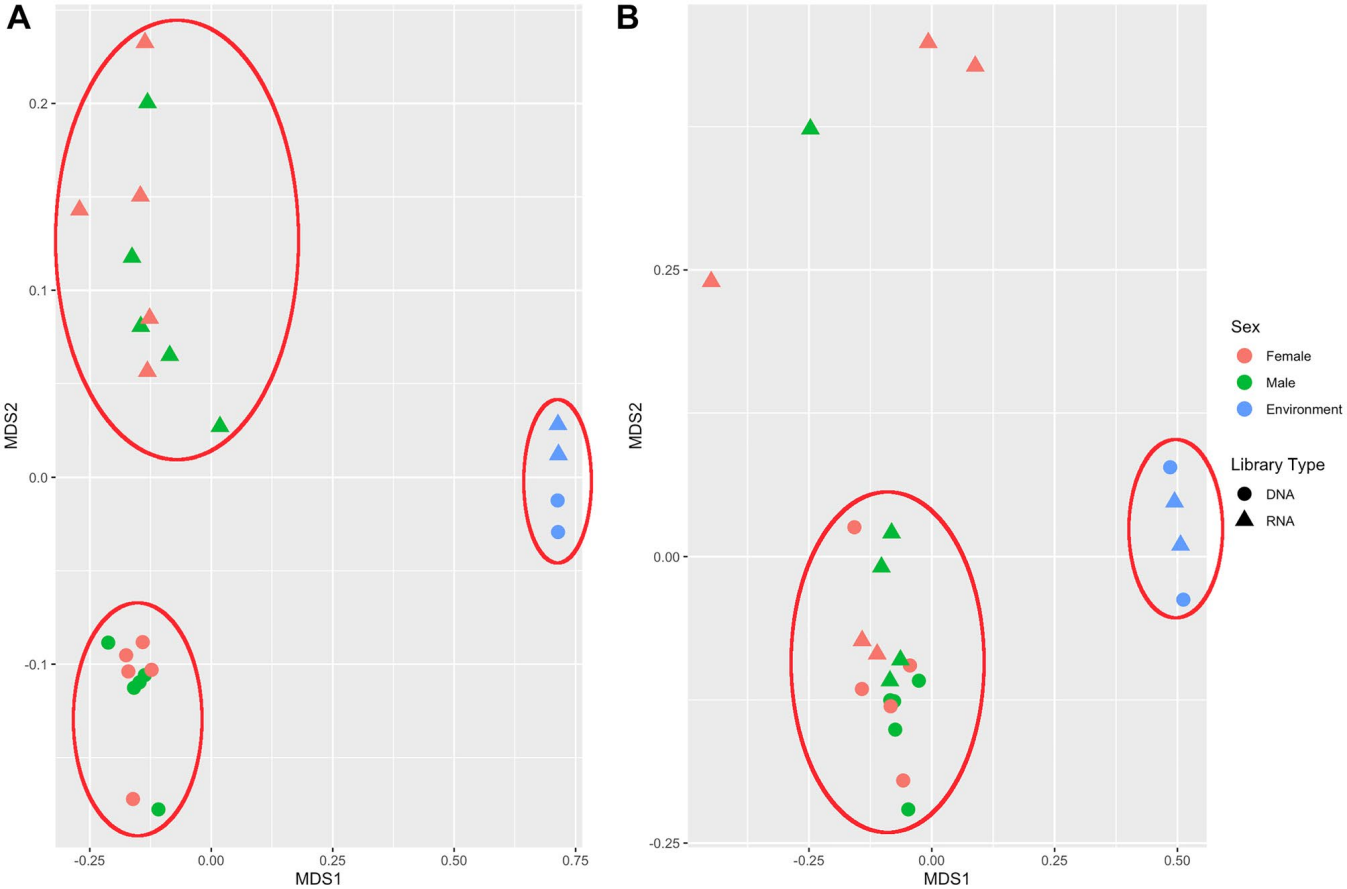
Composition of (**A**) bacterial and (**B**) archaeal community, displayed by non-metric multidimensional scaling (NMDS). Clustering samples are encircled. Colour code: green—male, red—female, blue—sea water. Filled circle—DNA samples, filled triangle—RNA samples.

Between sexes, significant differences were only observed for the composition of the active archaeal communities (p_cDNA_ = 0.015), but not for the other sample sets (present bacterial/archaeal communities; active bacterial communities). Further, differences between present (DNA-derived) and active (cDNA-derived) epibiotic communities were significant for bacteria, but not archaea (p_Bacteria_ = 0.003, p_Archaea_ = 0.203). Hence, the difference between active and present community was more pronounced than the difference between sexes. Non-metric multidimensional scaling (NMDS) highlights the relative similarity of bacterial epibiotic samples within the DNA and cDNA datasets and the dissimilarity between the two datasets (Fig. 5A). In contrast, epibiotic archaeal DNA and cDNA samples form one dense cluster, which indicates a rather high degree of similarity (Fig. 5B). Four archaeal cDNA samples are displayed very distant from the main cluster, most likely due to highly abundant sequences occurring exclusively in individual samples (Fig. 5B). NMDS further emphasises distinct differences between epibiotic and sea water samples for both bacterial and archaeal datasets (Fig. 5A, B).

### Major microbial taxa on *M. alvisca* and in ambient sea water

#### Bacterial community composition

**Figure 6:**
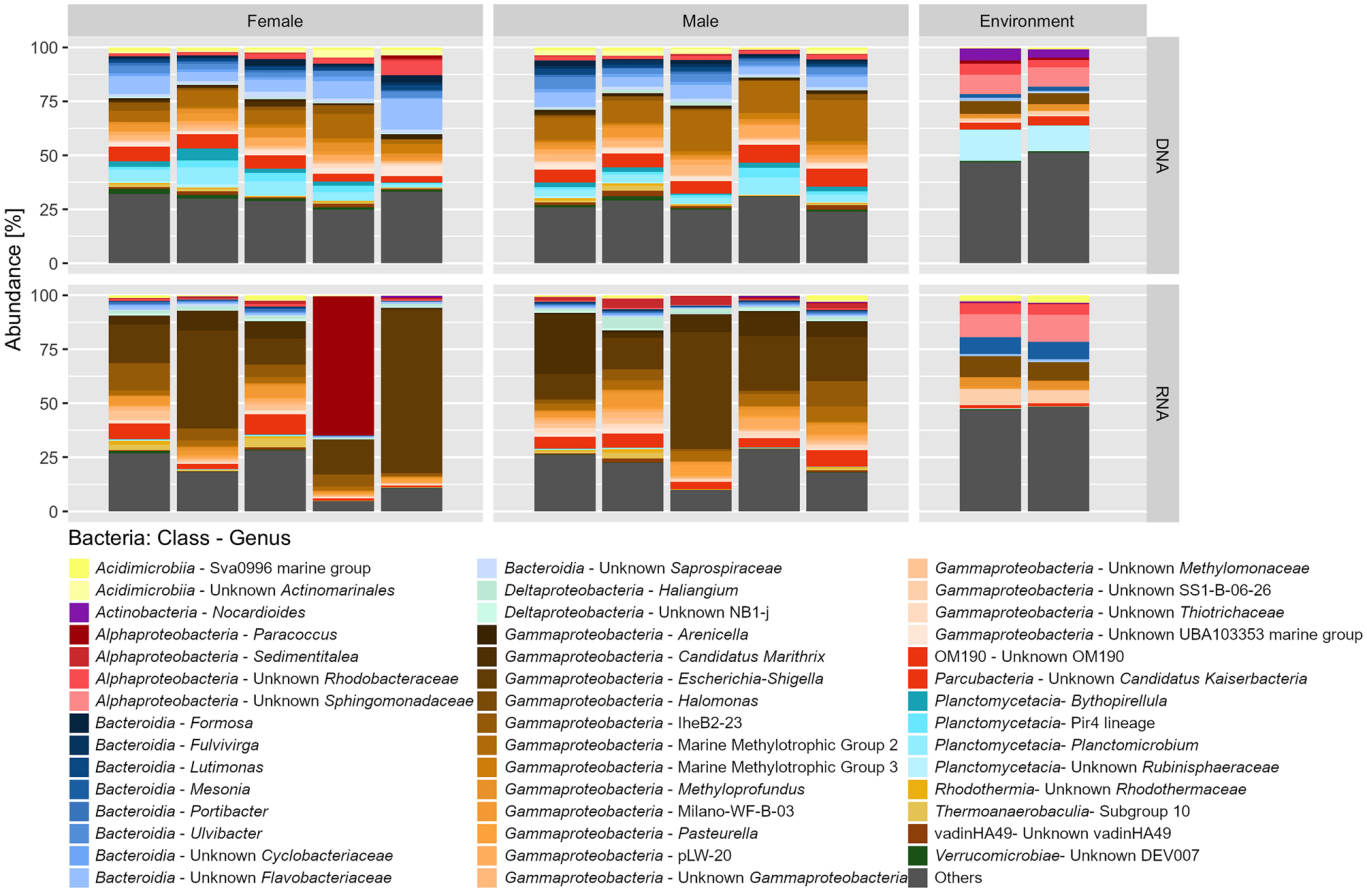
Abundance of bASVs in sea water and *M. alvisca* samples, displayed on genus-level or, if that was not possible, on the next identifiable superordinated rank. Others: taxa with < 0.5% abundance.

Carapaces of the *M. alvisca* specimens as well as the sea water samples were clearly dominated by the three major bacterial phyla *Proteobacteria*, *Planctomycetes* and *Bacteroidia*, together accounting for 70–80% of the entire bacterial community (Fig. 6, Supplement T3–4). Their most prominent representatives are highlighted in the following, percentages refer to the share the respective taxon constitutes in a given sample set. Taxonomy was assigned against a curated SILVA 132 database^26^.

In all samples, *Proteobacteria* was the most abundant (35–54%) phylum with the highest number of rRNA transcripts (60–95%). *Gammaproteobacteria* (DNA > 40%, cDNA > 75%) were dominant in *M. alvisca* samples, and to a lesser degree in the sea water samples (DNA > 15%, cDNA > 25%). On lower taxonomic ranks, *Methylomonaceae* and *Methylococcaceae* were the most abundant gammaproteobacterial families, accounting for almost one third of the epibiotic community in individual *M. alvisca* samples (Fig. 6). In contrast, these two families played little to no role in the bacterial community in sea water samples. *Beggiatoacea* (*Gammaproteobacteria*) were only present on *M. alvisca* (abundance < 3%) and not in sea water samples.

In sea water samples, *Proteobacteria* were represented mostly by *Alphaproteobacteria* (DNA and cDNA 24–29%). In *M. alvisca* samples, *Alphaproteobacteria* represented the second most abundant class (< 10%) and they additionally made up between 2.5 and 9.2% of total 16S rRNA gene transcripts in this sample type. The *Alphaproteobacteria* were mostly represented by the family *Rhodobacteraceae* that accounted for 2–3% of the DNA sequences in the epibiotic communities and for 5% in sea water. While their share of the cDNA sequences in sea water was > 5%, they constituted > 1% in only one out of ten *M. alvisca* samples. However, one specimen exhibited exceptionally high shares of *Rhodobacteraceae* (64% of rRNA transcripts), mainly attributable to the genus *Paracoccus* (Fig. 6). In sea water samples, unclassified *Sphingomonadaceae* (*Alphaproteobacteria*) were found in both DNA (< 10%) and cDNA (> 10%) datasets but were not detected in *M. alvisca* samples.

Interestingly, while no *Enterobacteriaceae* (*Gammaproteobacteria*) were detected in any of the DNA samples, the family was strongly represented in epibiotic cDNA. In individual samples, bASVs affiliated with *Escherichia-Shigella* contributed > 70% of all 16S rRNA gene transcripts (Fig. 6). In contrast, no 16S rRNA transcripts of *Escherichia-Shigella* were detected in sea water samples. We consider these highly abundant bASVs as true members of the epibiotic communities since we tested our primer sequences against the complete target sequences of the *Escherichia-Shigella* organisms obtained from NCBI to exclude discrimination and mismatching errors during the transcription of RNA into cDNA, but sequences matched completely. Moreover, negative controls (chemicals only) implemented in extraction, transcription and amplification did not show any contamination.

In all DNA and cDNA samples, *Bacteroidetes* and *Planctomycetes* constituted the second and third most dominant bacterial phyla, respectively (Fig. 6). They were mostly represented by *Bacteroidia* and *Planctomycetacia*, with *Bacteroidia* being detected almost two times more abundant on *M. alvisca* samples than in sea water. The majority of bASVs affiliated with *Bacteroidia* were *Flavobacteriaceae* and split into eight main genera. None of them was found in all three sample types (male/female/sea water), and the genera *Formosa* and *Lutimonas* were exclusively found in samples from female *M. alvisca* specimens. Flavobacterial genera contributed < 1–6% of DNA sequences and either zero or < 1% of cDNA sequences, except for *Mesonia* in sea water samples (RNA transcripts: ≤ 8%). In epibiotic DNA samples, *Planctomycetacia* were mainly represented by the genus *Planctomicrobium* (*Rubinisphaeraceae*) and several genera belonging to the *Pirellulaceae*, whereas this family occurred in minor proportions (mostly < 0.5%) in cDNA samples. In sea water samples, ASVs matching with *Pirellulaceae* were well-represented in the DNA sequences but showed very low cDNA frequencies and were not further characterised on genus level.

The fourth most abundant phylum in the DNA dataset, *Actinobacteria*, accounted for 2–8% and 6% of the bacterial sequences from *M. alvisca* samples and sea water, respectively and in all cDNA samples *Actinobacteria* accounted for up to 7%. *Actinobacteria* in *M. alvisca* samples were mainly attributed to *Acidimicrobia*, and to the class *Actinobacteria* in sea water samples.

#### Archaeal community composition

**Figure 7:**
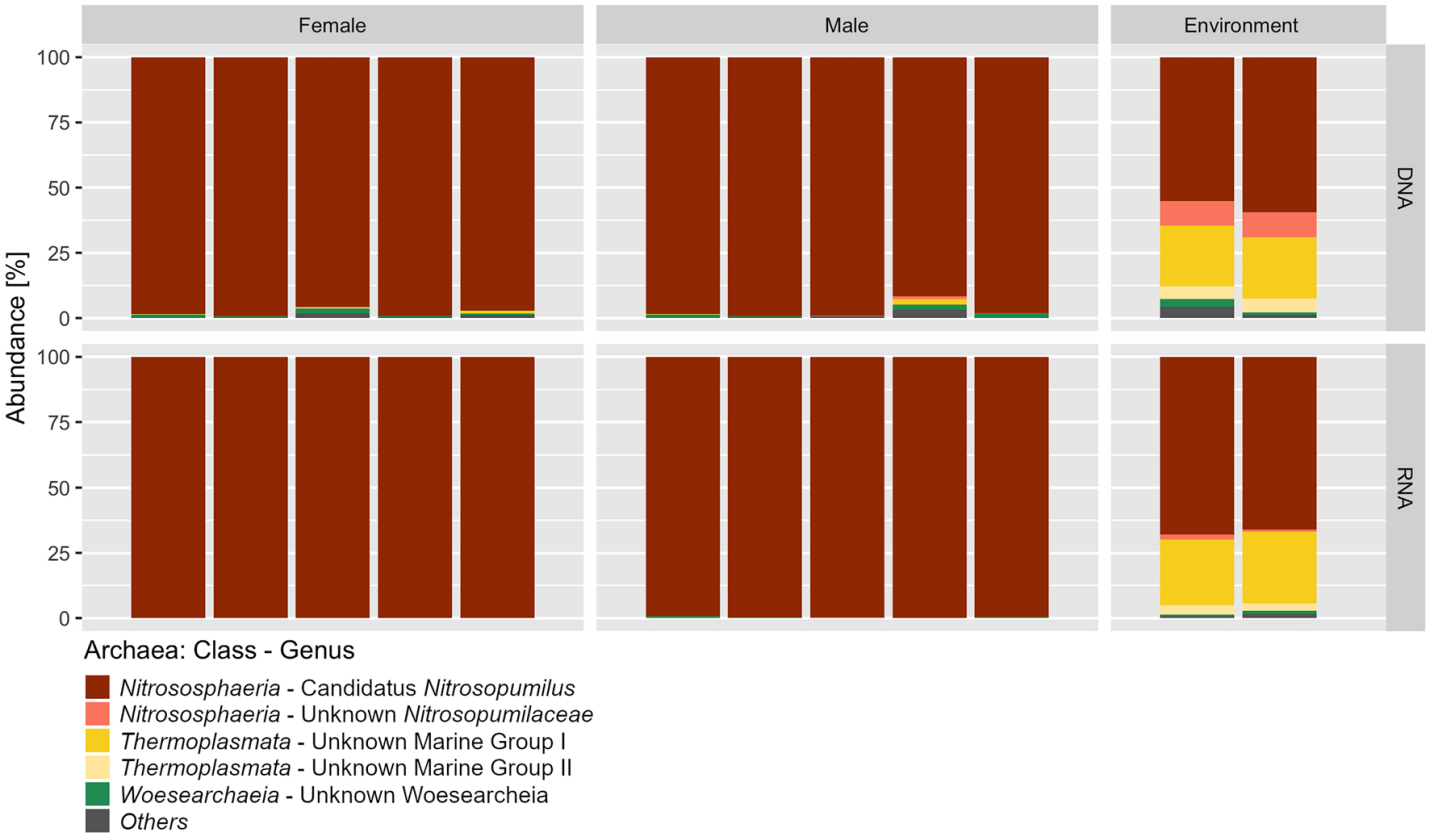
Abundance of aASVs in sea water and *M. alvisca* samples, displayed on genus-level or, if that was not possible, on the next identifiable superordinated rank. Others: taxa with < 0.5% abundance.

Epibiotic archaeal sequences were almost completely (DNA and cDNA 98–99.8%) affiliated with *Ca. Nitrosopumilus* (*Nitrososphaeria*, *Thaumarchaeota*) (Fig. 7; Supplement T5). Sequences affiliated with *Woesearchaeia* (*Nanoarchaeaota*) and Marine Group II (MGII, *Thermoplasmata*, *Euryarchaeota*) were the second and third most frequently detected on class level (Fig. 7). However, both groups together represented only 0.04% of all 16S rRNA gene transcripts.

Sea water samples were also dominated by *Nitrososphaeria*, most strongly represented by *Ca. Nitrosopumilus*. In contrast to > 90% *Nitrososphaeria* in the epibiotic dataset, this class accounted for only 64–68% of the archaeal DNA sequences in sea water. Second and third most abundant in sea water were also MGII and *Woesearchaeia*, constituting 28–29% and 3–8%, respectively (Fig. 7). In addition, aASVs representing Marine Group III (MGIII, *Thermoplasmata*, *Euryarchaeota*) made up 5% of the free-living archaeal sequences, but they were absent from the epibiotic dataset. The cDNA dataset showed a similar composition, with *Nitrososphaeria* accounting for two thirds of the sequences. *Woesearchaeia* were less abundant in cDNA compared to DNA, while *Thermoplasmata* accounted for almost one third of archaeal cDNA sequences in sea water samples.

### Global distribution and habitat diversity of main microbial taxa found on *M. alvisca* specimens

In the archaeal dataset (DNA and cDNA), eight aASVs were identified as the most abundant epibiotic aASVs (criteria: found in ≥ 70% of all *M. alvisca* samples with ≥ 0.95% abundance) and were subjected to phylogenetic analysis (Supplement F1, T6). Phylogenetic trees calculated with the most abundant ASVs and their best nBLAST hits are presented in Supplement F1 and F2. The dataset comprises of eight *Nitrosopumilus*-related sequences of which aASV_8 appeared in the sea water samples as well, although at lower abundance (0.37%). Only aASVs from the DNA dataset matched the defined criteria, due to the high microdiversity within the archaeal cDNA dataset. The bacterial dataset consisted of 25 bASVs that matched the criteria stated above. They were affiliated with the groups *Acidimicrobiia* (1 bASV), *Bacteroidia* (6 bASVs), *Planctomycetes* (4 bASVs), *Alphaproteobacteria* (1 bASV) and *Gammaproteobacteria* (13 bASVs) (Supplement F2, T7). The latter class was dominant in all compared sample sets (DNA/cDNA, male/female). None of these bASVs was found in sea water samples. BLAST-analysis revealed high similarities between our epibiotic archaeal and bacterial ASVs and sequences from various geographic locations. The distribution of the sequences related to those from *M. alvisca* included samples from the Mid Atlantic Ridge, European coastal waters, the Mediterranean Sea, the East and West Pacific, as well as Arctic and Antarctic waters. The respective habitats were often influenced by hydrothermal activity, cold or methane seeps or hydrocarbon and oil spills (Supplement F1, F2). In addition, we found many similarities with bacterial and archaeal sequences originating from sponges, corals, tube worms, fish and other invertebrates from different habitats and geographic locations. Further, bASV_14 and bASV_113 shared a ≥ 98% sequence similarity with sequences from the setae of the deep-sea crab *Shinkaia crosnieri*.

## Discussion

Despite the large distribution of *Munidopsis* spp. in global deep-sea environments, detailed information on their ecological role and interactions is scarce^27^. Here, we present the first comprehensive study on epibiotic microbial communities of the genus *Munidopsis* (Decapoda) and the first record of intraspecific genetic divergence within the species *Munidopsis alvisca*. Since the 1980s, *M. alvisca* specimens have been sighted and collected at different locations in the Guaymas Basin, including mainly hydrothermal vent and cold seep sites, but also abyssal plains^17,28–31^. However, many reports of *Munidopsis* spp. were often based solely on morphological identification from photographs taken in situ^32^. Considering the minute morphological differences by which some *Munidopsis* spp. are distinguished, the occurrence of misclassifications cannot be ruled out^29^. Consequently, sightings of *M. alvisca* have been questioned and revaluated over the past years^33^. Nowadays, traditional morphological identification is corroborated by molecular biological methods, allowing not only an integrative classification, but also investigation of phylogenetic relationships^18^. For instance, local morphological variants can be identified, by comparing specimens from different geographic locations that genetically belong to the same species. This might even allow for the discovery of recent or ongoing speciation^32^, or the identification of cryptic species^18^. As indicated by previous, comprehensive studies on phylogenetic relationships within the genus *Munidopsis*^18,32,34^, adequate sample size is crucial to this endeavour. To date only a single *M. alvisca* COI sequence was deposited in GenBank (MZ197621), which did not enable investigation of intraspecific divergence across habitats via comparison with our samples. Hence, our records provide a basis for future phylogenetic studies on *M. alvisca* in relation to other *Munidopsis* species.

The significant differences found between epibiotic and free-living microbial communities hint at an origin of epibionts other than the ambient sea water. Distinct differences between free-living and decapod-associated microbial communities have been highlighted previously^3^. An alternative source of epibionts often suggested for deep-sea hydrothermal vent crustaceans like *R. exoculata*, *K. hirsuta* or *S. crosnieri* is the horizontal acquisition from conspecifics^9,10,35,36^. This coincides with our findings that microbial communities found on all *M. alvisca* specimens were highly similar (Fig. 6). Several authors further assumed that specific epibionts are selected by the crustacean host via recognition of microbial cell surface modifications by the host immune system^37–40^. Other transmission routes include the vertical transfer from parent to offspring^16,40^, or acquisition from other locations (migration), which is facilitated in the Guaymas Basin, as different habitats are not strictly separated by biogeographic barriers^4^. We did not find significant differences between epibiotic communities on male and female specimens, which coincides with a previous report^41^. The authors compared the microbial community on 48 male and female (sex ratio 1:1) Chinese mitten crabs (*Eriocheir sinensis*, Decapoda). Where feasible, a combination of molecular biological studies on epibiotic community composition and observations of host behaviour might provide additional insights. Important factors, like migratory behaviour, which may differ between male and female decapods^41^, could be revealed and evaluated for impact on epibiotic community composition. Further, other environmental samples i.e. sediment or components of the chimney precipitates and plumes could be included in the comparison of free-living and host-associated microbial communities to identify the potential source of *M. alvisca* epibionts.

On *M. alvisca* carapaces we found an archaeal community that almost exclusively consists of aASVs affiliated with *Ca. Nitrosopumilus* (*Thaumarchaeota*). Members of this genus are known mainly as ammonia oxidisers^42^. Hence, we assume, that ammonia oxidation is one of the major metabolic processes carried out within the archaeal epibiotic community on *M. alvisca*. Frequently, *Nitrosopumilus* spp. have been reported in hydrothermal vent and other deep-sea environments, including sediments and hydrothermal plumes of the Guaymas Basin^15,43^. While associations of *Nitrosopumilus* spp. with hydrothermal vent sponges and corals are documented in the literature^42^, our study is the first to report *Ca. Nitrosopumilus* from a hydrothermal vent decapod. Only few previous attempts were made to study archaeal epibiotic communities on hydrothermal vent crustaceans and none of them yielded any sequences^44,45^. In the ambient sea water, we also found *Ca. Nitrosopumilus* in high relative abundances, however much less dominant in comparison to epibiotic communities. Hence, association with a host seems to be preferable for these microorganisms. One reason may be that the proximity of *M. alvisca* to the ammonia-enriched sediments and hydrothermal plume ensures direct access to substrate for chemosynthesis. Further, exposure to water mixing, dilution and resulting chemical alteration of fluid components, i.e. substrates, is prevented^14,42^. Vice versa, we assume that the increased abundance and activity of MGII and MGIII archaea in the water column may be related to the availability of sinking particulate organic matter (POM)^46^. However, little is known about the ecology of these two taxa in the deep-sea and information on metabolic capacities mainly originate from metagenomic studies^46^. As reported previously, MGII archaea show an affinity to particles and are prolific degraders of proteins, lipids and other macromolecules^47^, which corroborates our hypothesis.

Within the bacterial epibiotic community on *M. alvisca* carapaces, we found several taxa known for their abilities to oxidise methane (*Methylococcaceae*^14^, *Methylomonaceae*^42^) sulphide (*Marithrix* (formerly *Thiotrix*, *Thiotrichaceae*)^48^) or ammonia (*Pirellulaceae*^49^). All of the latter components occur in elevated concentrations in hydrothermal plumes at Rebecca’s Roost^1,15^ and their removal by microbial oxidation could contribute to the survival of *M. alvisca*. Sulphide oxidisers have been found frequently on other hydrothermal vent crustaceans, like *Alvinocaris longirostris*, *Rimicaris exoculata* and *Shinkaia crosnieri*^7,9,45^. Associations with sulphide “detoxifiers” are favourable for macro- and megafauna in sulphide-rich environments^45^, as many organisms suffer at elevated sulphide concentrations due to the inhibition of respiratory processes^10^. In return, as explained for *Nitrosopumilus*, the epibiotic partners are provided with a stable habitat in immediate proximity to the chimney. Also *Methylococcaceae* are frequently reported as ecto- and endosymbionts of deep-sea decapods^3,7,9^. In addition, the growth-promoting impact of methane-oxidising gill symbionts on their host, the hydrothermal vent mussel *Bathymodiolus childressi*, has been determined experimentally^50^. In the framework of this study, it remains speculative whether methane oxidisers provide direct advantages for *M. alvisca*. However, as previous studies have identified bacterial biofilms as a relevant food source of *Munidopsis* spp. and *M. alvisca* in particular^31,51^, we assume that epibiotic microbes to contribute to the *M. alvisca* diet.

In our study, heterotrophic bacterial degraders of labile organic matter, peptides, lipids and other high molecular weight compounds, like *Flavobacteriaceae, Rhodobacteraceae* and *Sphingomonadaceae*, were enriched in the sea water relative to the epibiotic community. As stated above, we assume that this is due to the availability of high amounts of POM in the water column. Members of the *Flavobacteriaceae* are known as major components of picoplankton and many representatives are specialised on the degradation of polysaccharides^52^. Moreover, all three families harbour several members that have been described to degrade (aromatic) hydrocarbons, crude oil or other xenobiotics^53–55^. This is reflected in our phylogenetic analysis of the most abundant bASVs, showing high sequence similarity with clones and isolates obtained from a variety of hydrocarbon- or oil-enriched habitats, highlighting the relevance of hydrocarbon degradation at Rebecca’s Roost. Extreme relative abundances of *Paracoccus* (*Rhodobacteraceae*) in our cDNA dataset may be due to locally increased concentrations of petroleum and hydrocarbons. Some members of the family, like *Paracoccus* are well-known as prolific degraders of those compounds^56^. Further, a rapidly rising dominance of *Roseobacter* clade members (marine *Rhodobacteraceae*^57^) has been found in response to the sedimentation of oil-rich POM after the Deepwater Horizon blowout in 2010^55^.

Our findings on free-living hydrothermal vent microbial communities are in accordance with previous studies^14,58^. In contrast, microbial communities in deep-sea, hydrothermal sediments share only few key members with epibiota identified in our study. While *Nitrosopumilaceae* and aerobic *Gammaproteobacteria* are still abundant in surface sediments that are influenced by sea water chemistry, their abundance rapidly decreases with depth^23^. After the depletion of oxygen in the first few millimetres to centimetres of sediment, thermophilic archaea, sulphate-reducing *Deltaproteobacteria* and other anaerobic taxa become dominant^2,23,59^. The benthic microbial community is hence strongly influenced by the availability of electron acceptors, as well as the increasing impact of heat and hydrothermal fluid components with depth^23,60^.

In contrast to previous studies, we found highly active bASVs affiliated with *Pasteurella* and *Escherichia-Shigella* on all *M.* *alvisca* specimens. As stated in the results section, all methodological and technical controls were free from contamination and allow the assumption that these taxa are true members of the bacterial epibiotic community. We hypothesise that they may originate from sloppy feeding, a behaviour which has been documented for other decapods^61^ and which results in the release of gut bacteria from prey animals. Even though information on marine members of *Pasteurella* and *Escherichia-Shigella* is scarce, other *Enterobacterales* have been found before in marine sediments and waters^62,63^. *Enterobacteriaceae* have been reported as epibionts of shallow-water sponges^64^, as intracellular pathogens of the hydrothermal vent mussel *Bathymodiolus azoricus* and as obligate biofilm-associates^65^. Despite their roles in anoxic environments where they reduce sulphate or thrive on fermentation, they may play a role in recycling of organic matter in oxic environments^66,67^, such as the carapace of *M. alvisca*. The observed discrepancies between activity and abundance of individual ASVs may be caused by one or several factors, including cell size, growth rate or nutrient availability. Depending on those aspects, RNA content can reach > 10 000 copies per cell^68^. We assume that the highly abundant cDNA sequences represent rare taxa with high metabolic activities that escape detection in the DNA samples due to their low abundances, which is not unusual for microbial assemblages in nutrient-restricted habitats^69,70^. Other authors have found 295 bASVs associated with sub-Arctic lichen that occur only in their cDNA dataset^71^. Most of these sequences were affiliated with *Proteobacteria* as well. Another study reports that a single bacterial species accounting only for 0.3% of the present community in a freshwater lake, constitutes 70% of the total carbon uptake^72^. Moreover, *Alpha*- and *Gammaproteobacteria* are known for their high growth rates which also leads to accumulation of RNA inside the cells and an enrichment relative to the DNA content^73^. *Pasteurella* or *Escherichia*-*Shigella* spp. might have gone undetected in previous investigations of decapod epibiota due to methodological or conceptual aspects. Most existing studies on epibiota of deep-sea crustaceans are based on clone libraries, FISH and morphological observations using electron microscopy and have limited their taxonomic resolution to the class level^74^. In addition, previous screenings have often focused on specific community members such as ammonia oxidisers^43^. However, to understand distribution and ecology of deep-sea microorganisms and observe distinct differences between habitats and hosts, a high taxonomic resolution and a holistic investigation of the microbial community are crucial^75^.

## Conclusion

By identification of all squat lobster specimens as *Munidopsis alvisca*, we demonstrated that Rebecca’s Roost, as many other hydrothermal vent environments, harbours a low-diversity, high-abundance decapod megafauna. We found a low intraspecific divergence, indicating no recent or ongoing diversification of the species at this location. However, future investigations should include collection and sequencing of *M. alvisca* specimens from other sites and in adequate numbers, to enable identification of local genetic variants. Microbial epibiotic communities on *M. alvisca* carapaces were dominated by the same taxa found abundant on other deep-sea hydrothermal vent decapods. We assume that *M. alvisca* benefits from sulphide detoxification by their epibiota, while attached microbes are supplied with a stable habitat in proximity to substrate-rich hydrothermal fluids. Hence, a mutualistic host-microbe relation is likely. The high microbial microdiversity, especially within cDNA samples from *M. alvisca* carapaces, indicates a high potential for rapid adaptation to minute changes in the immediate environment of epibiotic communities. Comparisons with literature further revealed that *M. alvisca* epibiotic communities, as well as sea water communities at Rebecca’s Roost are dominated by different taxa than benthic communities in the Guaymas Basin. Due to significant differences between attached and free-living microbial communities, we consider it unlikely that *M. alvisca* epibionts are acquired from ambient sea water. Possibly, epibionts are transferred between conspecifics or obtained from other environmental sources like sediment or precipitate surfaces which should be included for comparison in future studies. To trace back the origin of *M. alvisca* epibionts an individual study would be neccessary including also different molting and age stages as well as eggs.

## Material and methods

### Sample site and collection of samples

Galatheid squat lobsters were collected in the Guaymas Basin (Gulf of California, Mexico) during the *Atlantis* cruise AT42. Samples were taken at the site Rebecca’s Roost (111°24.42`W, 27°00.67`N). Animals were captured with the slurp gun of the submersible *Alvin* (dive 4995) in 1996 m depth from rocky precipitates surrounding a hydrothermal vent. Water samples were collected with Niskin bottles from the same site (approx. 0.2–0.5 m next to animals). None of the animals survived the ascent to the sea surface. Directly upon surfacing, the specimens were rinsed with filter-sterilised artificial sea water to remove loosely attached microbial cells. Swab samples were taken from the central part of the carapace and stored (4 °C overnight, − 80 °C afterwards) in RNAlater. To collect free-living microbial cells, one litre sea water was filtered through a 0.2 µm filter, which was frozen immediately. All samples, including the squat lobster specimens, were stored at − 80 °C until further use.

### Morphological identification of squat lobsters

The collected specimens were identified to species level at the German Centre for Marine Biodiversity Research (DZMB, Wilhelmshaven, Germany). Identification was achieved using taxonomic keys and original descriptions^20,30,76^. The sex was determined based on the presence of the first and second pairs of pleopods and the presence of the second up to the fifth pairs of pleopods in males and females, respectively, as well as the position of gonopores on the basis of pereopods in females and males, respectively. After identification, specimens were archived at DZMB and at the Institute for Chemistry and Biology of the Marine Environment (ICBM, Oldenburg, Germany). Collection data and photographs for each specimen are featured in the dataset Munidopsis_alvisca submitted in BOLD (http://dx.doi.org/10.5883/DS-MUNA).

### Molecular identification of squat lobsters

#### DNA extraction, amplification and sequencing

For species delimitation, the COI gene was analysed. Therefore, genomic DNA was extracted from abdominal or pereopod tissue from each specimen using 30 μl Chelex (InstaGene Matrix, Bio-Rad), according an established protocol^77^. A 658 bp fragment was amplified by polymerase chain reaction (PCR). PCR was performed as given in Supplement T5, using the primer pair jgLCO1490f and jgHCO2198r^78^, tailed with M13F (5′-TGTAAACGACGGCCAGT-3′) and M13R-pUC (5′-CAGGAAACAGCTATGAC-3′), respectively^78^. PCR products were purified using ExoSap-IT (Thermo Fisher Scientific, Waltham, MA, USA). The amplified fragments were sequenced in both directions at Macrogen Europe Laboratory (Amsterdam, The Netherlands).

#### Processing of COI sequences

Forward and reverse sequences from each specimen were assembled using Geneious v.9.1.7 (www.geneious.com). The assembled COI sequences were aligned using MAFFT v7.309^79^ and compared with sequences from other vent-associated *Munidopsis* species available in GenBank (Supplement T1). Sequence divergences (intra- and interspecific) were estimated as uncorrected p-distances using MEGA7. A neighbour-joining tree based on the COI sequences was constructed in MEGA7 using a p-distance substitution model, treating gaps and missing data with “pairwise deletion” and by running 1000 bootstrap replicates. Assembled COI sequences from this study were deposited in GenBank (MW591339-MW591351).

### Microbial nucleic acid extraction and amplification

#### Nucleic acid extraction and purification

All chemicals used during the nucleic acid extraction were RNAse-free, devices were cleaned thoroughly with RNAse AWAY before use. An extraction blank (chemicals only) was included to ensure purity of used chemicals. DNA and RNA were extracted simultaneously following a phenol–chloroform protocol (modified after^80^, cf. Supplementary Information). The extracted RNA was further purified using the RNeasy Mini Kit (Qiagen, Hilden, Germany). Residual genomic DNA was removed with the Turbo DNA-free kit (Invitrogen, Carlsbad, USA) before the purified RNA was transcribed into cDNA using the SuperScript Double-Stranded cDNA synthesis kit (Invitrogen, Carlsband, USA). All commercial kits were applied according to the manufacturer’s instructions.

#### Amplification and purification of archaeal and bacterial DNA and cDNA for Illumina sequencing

The 16S rRNA gene of bacterial and archaeal DNA and cDNA was amplified using the primer pair of^81^ for bacteria, and the primers 340f^82^ and 840r^83^ for archaea. Sequences of Illumina adapters for both target organisms were 5′-TCGTCGGCAGCGTCAGATGTGTATAAGAGACAG (forward) and 3′-GTCTCGTGGGCTCGGAGATGTGTATAAGAGACAG (reverse). PCR settings are given in Supplement T6, T7. For each sample three replicate PCRs were performed to minimise PCR bias. The triplicates were pooled before purification with the QIAquick PCR purification kit (Qiagen, Hilden, Germany) and sent to the Institute of Microbiology and Genetics (University of Göttingen, Germany) for sequencing.

### Amplicon sequencing of archaeal and bacterial 16S rRNA genes

Indices and Illumina sequencing adapters were attached to microbial PCR products using the Nextera XT Index kit (Illumina, San Diego, CA, USA). Index PCR was performed with 5 µl template, 2.5 µl of each index primer, 12.5 µl of 2 × KAPA HiFi HotStart ReadyMix and 2.5 µl PCR-grade water. Thermal cycling was as follows: 95 °C for 3 min, 8 cycles of 30 s at 95 °C, 30 s at 55 °C and 30 s at 72 °C, final extension at 72 °C for 5 min. Quantification of the products was performed using a Qubit fluorometer (Invitrogen GmbH, Karlsruhe, Germany). MagSi-NGSPREP Plus Magnetic beads (Steinbrenner Laborsysteme GmbH, Wiesenbach, Germany) were used for purification of the indexed PCR products and normalization was performed with the Janus Automated Workstation (Perkin Elmer, Waltham, MA, USA). Sequencing was conducted on the Illumina MiSeq platform with dual indexing and MiSeq reagent kit v3 (600 cycles). If not stated otherwise, kits were used according to the manufacturer’s instructions.

### Processing of microbial amplicon sequences

Based on the primers, samples were split into a bacterial and an archaeal dataset and analysed separately. Both datasets were processed in parallel with the same workflow. Initially, primer sequences were trimmed with cutadapt, discarding any sequence pairs lacking primer sequences. Up to 20% mismatches within the primer sequence were allowed to retain amplicons with single mismatches. Sequences were processed within QIIME 2^84^. Reads were trimmed to remove low quality ends and filtered according to overall read quality. Filtered reads were processed within QIIME using the dada2 plugin, which calculates run-specific error models and denoises sequences according to the latter. Denoised sequences were merged and checked for chimeric reads. Resulting ASVs were classified using the Naïve Bayesian classifier, implemented in the classify-sklearn plugin of QIIME 2, against a curated SILVA 132 database^26^, which was fitted to the primer region of the respective dataset. Finally, reads were exported into count tables for further statistical analysis. ASVs with less than five counts were excluded from the data. Sequencing data is deposited in ENA (European Nucleotide Archive) under the accession PRJEB46145.

### Statistical analysis

Prokaryotic communities were visualised with barplots. For diversity measures, samples were rarefied to the lowest common count number before calculating richness, evenness and Shannon index using the R package vegan^85^. We used NMDS to display sample variability within each dataset. Therefore, count tables were transformed into Bray–Curtis distances and NMDS was calculated using the *metaMDS* function from vegan. Read data was standardised using Hellinger-transformation before inferring Bray–Curtis-Distance. Bray–Curtis distances were then tested against the sex of the host for various group combinations (DNA, RNA, Environment) by using a permutational MANOVA (function *adonis,* vegan package). Significant effects occurring in cases with high group dispersion were judged by inspecting their mean differences in relation to internal group variation. Beta-diversity was calculated as distance to group centroids using *betadisper* function from the vegan package. Significant differences between beta-diversity of groups were inferred from ANOVA. Venn-diagrams were used to display ASVs shared between the different sexes or the habitat. All analyses were conducted within the R computing environment^86^. Heatmaps displaying microdiversity within taxonomic groups were calculated using features of the *pheatmap* package. For display, ASVs were selected that were present in an abundance > 0.001% and (i) were present in at least one sample at a relative abundance > 1% or (ii) were present in at least 2% of samples at a relative abundance > 0.1% or (iii) were present in at least 5% of samples at any abundance level and (vi) minimum of 1000 counts per sample.

### Phylogenetic analysis of amplicon sequences

The most abundant ASVs in the archaeal and bacterial datasets were subjected to phylogenetic analysis via nBLAST comparison with closely related sequences in the GenBank database. ASVs were selected for comparison according to the following criteria: The most abundant ASVs of one *M. alvisca* dataset (bacteria OR archaea AND DNA OR cDNA AND male OR female) that were found in at least 70% of the respective sample type (environment OR animals) and make up ≥ 0.95% of the respective community. Neighbor-joining trees were calculated using the ARB software package^87^. Trees were calculated with 1000 replicates using only sequences ≥ 1200 bp. Further sequences were added subsequently to the existing tree.

## Supplementary Information

Supplementary Information.

## Data Availability

The datasets generated and analysed in the current study are available in the following repositories: Sequences, trace files, collection data and photographs of each *Munidopsis alvisca* specimen are listed in the datasets Munidopsis_alvisca in BOLD (http://dx.doi.org/10.5883/DS-MUNA). *M. alvisca* COI sequences are further available from GenBank (MW591339-MW591351). Microbial sequencing data is deposited in ENA (European Nucleotide Archive) under the accession number PRJEB46145.
