# Supplementary material for: Microbial epibiotic community of the deep-sea galatheid squat lobster Munidopsis alvisca
Source: Sci Rep. 2022 Feb 17;12:2675. doi: 10.1038/s41598-022-06666-x (PMC8854721; doi:10.1038/s41598-022-06666-x)
Supplement: Supplementary file 1 — Supplementary Information. [file 41598_2022_6666_MOESM1_ESM.docx]

**Supplementary Material**

## Taxonomic identification of *Munidopsis alvisca* Williams, 1988

Supplement T 1: Mean nucleotide distances (p-distance) of cytochrome c oxidase I (COI) gene sequences within *Munidopsis* *alvisca* specimens (this study) and in comparison with other *Munidopsis* hydrothermal vent species (references for sequences given in table).

|  |  | *M. alvisca* | *M. alvisca* | *M. alvisca* | *M. alvisca* | *M. alvisca* | *M. alvisca* | *M. alvisca* | *M. alvisca* | *M. alvisca* |
| --- | --- | --- | --- | --- | --- | --- | --- | --- | --- | --- |
|  |  | MW591349 | MW591348 | MW591343 | MW591342 | MW591340 | MW591350 | MW591347 | MW591346 | MW591339 |
| *M. alvisca* | MW591349 |  |  |  |  |  |  |  |  |  |
| *M. alvisca* | MW591348 | 0.0076 |  |  |  |  |  |  |  |  |
| *M. alvisca* | MW591343 | 0.0046 | 0.0061 |  |  |  |  |  |  |  |
| *M. alvisca* | MW591342 | 0.0061 | 0.0076 | 0.0015 |  |  |  |  |  |  |
| *M. alvisca* | MW591340 | 0.0061 | 0.0076 | 0.0015 | 0.0030 |  |  |  |  |  |
| *M. alvisca* | MW591350 | 0.0076 | 0.0030 | 0.0030 | 0.0046 | 0.0046 |  |  |  |  |
| *M. alvisca* | MW591347 | 0.0076 | 0.0030 | 0.0030 | 0.0046 | 0.0046 | 0.0000 |  |  |  |
| *M. alvisca* | MW591346 | 0.0061 | 0.0046 | 0.0015 | 0.0030 | 0.0030 | 0.0015 | 0.0015 |  |  |
| *M. alvisca* | MW591339 | 0.0091 | 0.0076 | 0.0046 | 0.0061 | 0.0061 | 0.0046 | 0.0046 | 0.0030 |  |
| *M. alvisca* | MW591344 | 0.0091 | 0.0076 | 0.0046 | 0.0061 | 0.0061 | 0.0046 | 0.0046 | 0.0030 | 0.0000 |
| *M. alvisca* | MW591345 | 0.0107 | 0.0061 | 0.0061 | 0.0076 | 0.0076 | 0.0030 | 0.0030 | 0.0046 | 0.0015 |
| *M. alvisca* | MW591351 | 0.0107 | 0.0061 | 0.0061 | 0.0076 | 0.0076 | 0.0030 | 0.0030 | 0.0046 | 0.0046 |
| *M. alvisca* | MZ197621^*^ | 0.0071 | 0.0124 | 0.0053 | 0.0071 | 0.0071 | 0.0088 | 0.0088 | 0.0071 | 0.0106 |
| *M. lauensis* | MH599070^**^ | 0.0289 | 0.0304 | 0.0244 | 0.0259 | 0.0259 | 0.0274 | 0.0274 | 0.0259 | 0.0289 |
| *M. lauensis* | KF774316^***^ | 0.0289 | 0.0304 | 0.0244 | 0.0259 | 0.0259 | 0.0274 | 0.0274 | 0.0259 | 0.0289 |
| *M. lauensis* | EF157850^****^ | 0.0289 | 0.0304 | 0.0244 | 0.0259 | 0.0259 | 0.0274 | 0.0274 | 0.0259 | 0.0289 |
| *M. recta* | DQ677695^*****^ | 0.1243 | 0.1281 | 0.1224 | 0.1205 | 0.1243 | 0.1243 | 0.1243 | 0.1224 | 0.1224 |
| *M. bracteosa* | DQ677684^*****^ | 0.1299 | 0.1337 | 0.1281 | 0.1262 | 0.1299 | 0.1299 | 0.1299 | 0.1281 | 0.1281 |
| *M. exuta* | DQ677690^*****^ | 0.1337 | 0.1375 | 0.1318 | 0.1299 | 0.1337 | 0.1337 | 0.1337 | 0.1318 | 0.1318 |
| *M. lentigo* | KY581547^******^ | 0.2443 | 0.2500 | 0.2443 | 0.2462 | 0.2443 | 0.2462 | 0.2462 | 0.2443 | 0.2424 |
| \| * \| Murdock S.A., Tunnicliffe V., Boschen-Rose R.E. & Juniper S.K. (2021). Emergent core communities of microbes, meiofauna and macrofauna at hydrothermal vents. ISME Communications, 1: 27 \| \| --- \| --- \| \| ** \| Li L. (unpublished data). Community structure and functional gene abundance analysis of epibiotic bacteria of squat lobsters (Munidopsis lauensis) in Pacmanus hydrothermal vent field \| \| *** \| Lin C.W., Tsuchida S., Lin S., Berndt C. & Chan T.Y. (2013). Munidopsis lauensis Baba & de Saint Laurent, 1992 (Decapoda, Anomura, Munidopsidae), a newly recorded squat lobster from a cold seep in Taiwan. Zootaxa 3737 (1): 92-96. \| \| **** \| Cubelio S.S., Tsuchida S., Hendrickx M.E., Kado R. & Watanabe S. (2007). A new species of vent associated Munidopsis (Crustacea: Decapoda: Anomura: Galatheidae) from the Western Pacific, with notes on its genetic identification. Zootaxa 1435: 25-36. \| \| ***** \| Jones W.J. & Macpherson E. (2007). Molecular phylogeny of the East Pacific squat lobsters of the genus Munidopsis (Decapoda, Galatheidae) with the description of seven new species. Journal of Crustacean Biology 27 (3): 477-501. \| \| ****** \| Goffredi S.K., Johnson S., Tunnicliffe V., Caress D., Clague D., Escobar E., Lundsten L., Paduan J.B., Rouse G., Salcedo D.L., Soto L.A., Spelz-Madero R., Zierenberg R. & Vrijenhoek,R. (2017). Hydrothermal vent fields discovered in the southern Gulf of California clarify role of habitat in augmenting regional diversity. Proceedings of the Royal Society: Biological Sciences, 284 (1859) \| | | | | | | | |  |  |  |

| **Supplement T 1 continued.** | | | | | | | |  |  |  |
| --- | --- | --- | --- | --- | --- | --- | --- | --- | --- | --- |
|  |  | *M. alvisca* | *M. alvisca* | *M. alvisca* | *M. lauensis* | *M. lauensis* | *M. lauensis* | *M. recta* | *M. bracteosa* | *M. exuta* |
|  |  | MW591344 | MW591345 | MW591351 | MH599070^*^ | KF774316^**^ | EF157850^***^ | DQ677695^****^ | DQ677684^****^ | DQ677690^****^ |
| *M. alvisca* | MW591349 |  |  |  |  |  |  |  |  |  |
| *M. alvisca* | MW591348 |  |  |  |  |  |  |  |  |  |
| *M. alvisca* | MW591343 |  |  |  |  |  |  |  |  |  |
| *M. alvisca* | MW591342 |  |  |  |  |  |  |  |  |  |
| *M. alvisca* | MW591340 |  |  |  |  |  |  |  |  |  |
| *M. alvisca* | MW591350 |  |  |  |  |  |  |  |  |  |
| *M. alvisca* | MW591347 |  |  |  |  |  |  |  |  |  |
| *M. alvisca* | MW591346 |  |  |  |  |  |  |  |  |  |
| *M. alvisca* | MW591339 |  |  |  |  |  |  |  |  |  |
| *M. alvisca* | MW591344 |  |  |  |  |  |  |  |  |  |
| *M. alvisca* | MW591345 | 0.0015 |  |  |  |  |  |  |  |  |
| *M. alvisca* | MW591351 | 0.0046 | 0.0030 |  |  |  |  |  |  |  |
| *M. alvisca* | MZ197621^*^ | 0.0106 | 0.0124 | 0.0106 |  |  |  |  |  |  |
| *M. lauensis* | MH599070^**^ | 0.0289 | 0.0304 | 0.0304 |  |  |  |  |  |  |
| *M. lauensis* | KF774316^***^ | 0.0289 | 0.0304 | 0.0304 | 0.0000 |  |  |  |  |  |
| *M. lauensis* | EF157850^****^ | 0.0289 | 0.0304 | 0.0304 | 0.0000 | 0.0000 |  |  |  |  |
| *M. recta* | DQ677695^*****^ | 0.1224 | 0.1243 | 0.1224 | 0.1149 | 0.1149 | 0.1149 |  |  |  |
| *M. bracteosa* | DQ677684^*****^ | 0.1281 | 0.1299 | 0.1281 | 0.1243 | 0.1243 | 0.1243 | 0.0169 |  |  |
| *M. exuta* | DQ677690^*****^ | 0.1318 | 0.1337 | 0.1318 | 0.1243 | 0.1243 | 0.1243 | 0.0188 | 0.0169 |  |
| *M. lentigo* | KY581547^******^ | 0.2424 | 0.2443 | 0.2443 | 0.2462 | 0.2462 | 0.2462 | 0.2201 | 0.2239 | 0.2258 |
| \| * \| Murdock S.A., Tunnicliffe V., Boschen-Rose R.E. & Juniper S.K. (2021). Emergent core communities of microbes, meiofauna and macrofauna at hydrothermal vents. ISME Communications, 1: 27 \| \| --- \| --- \| \| ** \| Li L. (unpublished data). Community structure and functional gene abundance analysis of epibiotic bacteria of squat lobsters (Munidopsis lauensis) in Pacmanus hydrothermal vent field \| \| *** \| Lin C.W., Tsuchida S., Lin S., Berndt C. & Chan T.Y. (2013). Munidopsis lauensis Baba & de Saint Laurent, 1992 (Decapoda, Anomura, Munidopsidae), a newly recorded squat lobster from a cold seep in Taiwan. Zootaxa 3737 (1): 92-96. \| \| **** \| Cubelio S.S., Tsuchida S., Hendrickx M.E., Kado R. & Watanabe S. (2007). A new species of vent associated Munidopsis (Crustacea: Decapoda: Anomura: Galatheidae) from the Western Pacific, with notes on its genetic identification. Zootaxa 1435: 25-36. \| \| ***** \| Jones W.J. & Macpherson E. (2007). Molecular phylogeny of the East Pacific squat lobsters of the genus Munidopsis (Decapoda, Galatheidae) with the description of seven new species. Journal of Crustacean Biology 27 (3): 477-501. \| \| ****** \| Goffredi S.K., Johnson S., Tunnicliffe V., Caress D., Clague D., Escobar E., Lundsten L., Paduan J.B., Rouse G., Salcedo D.L., Soto L.A., Spelz-Madero R., Zierenberg R. & Vrijenhoek,R. (2017). Hydrothermal vent fields discovered in the southern Gulf of California clarify role of habitat in augmenting regional diversity. Proceedings of the Royal Society: Biological Sciences, 284 (1859) \| | | | | | | | |  |  |  |

## Microbial diversity: Diversity indices

Supplement T 2: Diversity indices of sequencing results obtained for sea water and *Munidopsis* *alvisca* samples expressed as Shannon index. For the calculation, all samples were rarefied to equal counts: 9167 for *Bacteria* and 5629 for *Archaea*. Marked in grey: samples that remained below the count threshold.

| **Sample** | **ID** |  | **Counts** | **Richness** | **Shannon** | **Evenness** |  | **Counts** | **Richness** | **Shannon** | **Evenness** | |
| --- | --- | --- | --- | --- | --- | --- | --- | --- | --- | --- | --- | --- |
| **Library type: DNA** | |  | **Archaea** | | | |  | **Bacteria** | | | |  |
| Male | 1m |  | 21389 | 29 | 2.08 | 0.62 |  | 19371 | 496 | 5.42 | 0.87 | |
| Male | 2m |  | 14141 | 23 | 1.86 | 0.59 |  | 50835 | 675 | 5.59 | 0.86 | |
| Male | 4m |  | 14446 | 24 | 1.98 | 0.62 |  | 17559 | 422 | 5.08 | 0.84 | |
| Male | 5m |  | 11138 | 49 | 2.39 | 0.61 |  | 14375 | 411 | 5.29 | 0.88 | |
| Male | 6m |  | 49112 | 32 | 1.84 | 0.53 |  | 24241 | 531 | 5.39 | 0.86 | |
| Female | 7m |  | 38464 | 40 | 1.88 | 0.51 |  | 24748 | 461 | 5.19 | 0.85 | |
| Female | 9m |  | 14888 | 24 | 1.95 | 0.61 |  | 17936 | 533 | 5.55 | 0.88 | |
| Female | 10m |  | 11166 | 70 | 2.46 | 0.58 |  | 14925 | 498 | 5.52 | 0.89 | |
| Female | 11m |  | 6207 | 26 | 1.68 | 0.52 |  | 22947 | 564 | 5.55 | 0.88 | |
| Female | 12m |  | 2058 | 15 | 1.89 | 0.70 |  | 14267 | 414 | 5.13 | 0.85 | |
| Sea water | RR1 |  | 35683 | 300 | 3.54 | 0.62 |  | 9167 | 173 | 4.20 | 0.81 | |
| Sea water | RR2 |  | 8639 | 99 | 3.12 | 0.68 |  | 10312 | 201 | 4.33 | 0.82 | |
|  |  |  |  |  |  |  |  |  |  |  |  | |
| **Library type: RNA** | |  |  |  |  |  |  |  |  |  |  | |
| Male | 1m |  | 13869 | 63 | 2.43 | 0.59 |  | 58248 | 573 | 4.38 | 0.69 | |
| Male | 2m |  | 13862 | 63 | 2.13 | 0.52 |  | 14428 | 356 | 4.91 | 0.84 | |
| Male | 4m |  | 5629 | 36 | 2.77 | 0.77 |  | 5645 | 100 | 2.54 | 0.55 | |
| Male | 5m |  | 984 | 30 | 3.04 | 0.89 |  | 15487 | 346 | 4.30 | 0.74 | |
| Male | 6m |  | 6398 | 79 | 2.88 | 0.66 |  | 19581 | 386 | 4.51 | 0.76 | |
| Female | 7m |  | 8593 | 42 | 1.69 | 0.45 |  | 14704 | 403 | 4.75 | 0.79 | |
| Female | 9m |  | 12406 | 105 | 2.64 | 0.57 |  | 12449 | 266 | 3.38 | 0.61 | |
| Female | 10m |  | 1451 | 30 | 3.04 | 0.89 |  | 19346 | 483 | 5.14 | 0.83 | |
| Female | 11m |  | 3219 | 23 | 1.10 | 0.35 |  | 27180 | 194 | 1.89 | 0.36 | |
| Female | 12m |  | 675 | 13 | 2.33 | 0.91 |  | 8679 | 133 | 1.90 | 0.39 | |
| Sea water | RR1 |  | 26240 | 235 | 3.98 | 0.73 |  | 7221 | 145 | 4.17 | 0.84 | |
| Sea water | RR2 |  | 15045 | 234 | 3.99 | 0.73 |  | 13087 | 202 | 4.25 | 0.80 | |

## Abundances of major bacterial and archaeal taxa

Supplement T 3: Share (in %) of each taxonomic unit listed below on the cDNA bacterial community found in sea water and *M. alvisca* carapace samples. These values are visualised in Figure 6. Taxa <0.5 % abundance are summarised in "others". cDNA - transcribed RNA; *Ca*. - *Candidatus*, Un. - unknown

|  |  |  | **cDNA** | | | | | | | | | | | |
| --- | --- | --- | --- | --- | --- | --- | --- | --- | --- | --- | --- | --- | --- | --- |
|  |  |  | **Female** | | | | | **Male** | | | | | **Sea water** | |
| **Class** | **Family** | **Genus** | **7m** | **9m** | **10m** | **11m** | **12m** | **1m** | **2m** | **4m** | **5m** | **6m** | **RR1** | **RR2** |
| *Acidimicrobiia* | *Microtrichaceae* | Sva0996 marine group | 1.2 | 0.5 | 2.2 | 0.2 | 0.3 | 0.6 | 1.1 | 0.0 | 0.2 | 2.1 | 2.8 | 3.4 |
| *Acidimicrobiia* | Unknown *Actinomarinales* | - | 0.2 | 0.1 | 0.4 | 0.3 | 0.1 | 0.2 | 0.5 | 0.2 | 0.1 | 1.0 | 0.0 | 0.0 |
| *Actinobacteria* | *Nocardioidaceae* | *Nocardioides* | 0.0 | 0.1 | 0.0 | 0.0 | 0.8 | 0.0 | 0.0 | 0.0 | 0.6 | 0.3 | 0.7 | 0.5 |
| *Alphaproteobacteria* | *Rhodobacteraceae* | *Paracoccus* | 0.1 | 0.1 | 0.2 | 64.3 | 0.1 | 0.1 | 0.0 | 0.0 | 0.5 | 0.2 | 0.3 | 0.3 |
| *Alphaproteobacteria* | *Rhodobacteraceae* | *Sedimentitalea* | 0.3 | 0.7 | 1.2 | 0.1 | 0.6 | 1.5 | 4.3 | 4.3 | 0.8 | 2.4 | 0.0 | 0.0 |
| *Alphaproteobacteria* | *Rhodobacteraceae* |  | 0.9 | 0.3 | 1.4 | 0.0 | 0.6 | 0.4 | 0.4 | 0.0 | 0.1 | 0.8 | 5.1 | 4.8 |
| *Alphaproteobacteria* | *Sphingomonadaceae* |  | 0.0 | 0.1 | 0.0 | 0.0 | 0.2 | 0.2 | 0.1 | 0.1 | 0.1 | 0.0 | 10.5 | 12.6 |
| *Bacteroidia* | *Flavobacteriaceae* | *Formosa* | 0.0 | 0.0 | 0.1 | 0.0 | 0.0 | 0.1 | 0.0 | 0.0 | 0.3 | 0.1 | 0.0 | 0.0 |
| *Bacteroidia* | *Cyclobacteriaceae* | *Fulvivirga* | 0.2 | 0.3 | 0.2 | 0.1 | 0.2 | 0.4 | 0.7 | 0.0 | 0.3 | 0.4 | 0.0 | 0.0 |
| *Bacteroidia* | *Flavobacteriaceae* | *Lutimonas* | 0.3 | 0.2 | 0.4 | 0.1 | 0.0 | 0.4 | 0.3 | 0.7 | 0.1 | 0.3 | 0.0 | 0.0 |
| *Bacteroidia* | *Flavobacteriaceae* | *Mesonia* | 0.0 | 0.0 | 0.0 | 0.0 | 0.0 | 0.2 | 0.1 | 0.0 | 0.0 | 0.0 | 7.8 | 8.2 |
| *Bacteroidia* | *Saprospiraceae* | *Portibacter* | 0.9 | 0.5 | 1.3 | 0.1 | 0.1 | 0.4 | 0.5 | 0.2 | 0.6 | 0.8 | 0.0 | 0.0 |
| *Bacteroidia* | *Flavobacteriaceae* | *Ulvibacter* | 0.4 | 0.2 | 0.4 | 0.2 | 0.0 | 0.3 | 0.2 | 0.2 | 0.5 | 0.2 | 0.0 | 0.0 |
| *Bacteroidia* | *Cyclobacteriaceae* | - | 0.4 | 0.4 | 0.5 | 0.2 | 0.1 | 0.5 | 0.8 | 0.1 | 0.3 | 0.4 | 0.1 | 0.1 |
| *Bacteroidia* | *Flavobacteriaceae* | - | 1.9 | 0.6 | 0.9 | 0.4 | 0.9 | 0.8 | 0.6 | 0.2 | 0.9 | 0.5 | 0.9 | 0.9 |
| *Bacteroidia* | *Saprospiraceae* | - | 0.5 | 1.2 | 0.4 | 0.2 | 0.1 | 0.2 | 0.5 | 0.3 | 0.1 | 0.5 | 0.0 | 0.0 |
| *Deltaproteobacteria* | *Haliangiaceae* | *Haliangium* | 1.1 | 1.5 | 1.2 | 0.4 | 1.1 | 0.9 | 5.0 | 2.4 | 0.5 | 1.3 | 0.0 | 0.0 |
| *Deltaproteobacteria* | Unknown NB1-j | - | 1.2 | 0.5 | 1.1 | 0.2 | 0.5 | 1.0 | 1.0 | 0.2 | 1.2 | 0.6 | 0.1 | 0.4 |
| *Gammaproteobacteria* | *Arenicellaceae* | *Arenicella* | 0.2 | 0.1 | 0.2 | 0.0 | 0.1 | 0.5 | 1.1 | 0.0 | 0.8 | 0.4 | 0.0 | 0.0 |
| *Gammaproteobacteria* | *Beggiatoaceae* | *Ca.* *Marithrix* | 3.8 | 8.9 | 7.9 | 0.3 | 0.9 | 27.7 | 2.3 | 8.3 | 10.9 | 6.8 | 0.0 | 0.0 |
| *Gammaproteobacteria* | *Enterobacteriaceae* | *Escherichia*-*Shigella* | 17.9 | 45.5 | 12.1 | 15.9 | 75.4 | 11.9 | 14.7 | 54.0 | 25.4 | 20.6 | 0.0 | 0.0 |
| *Gammaproteobacteria* | *Halomonadaceae* | *Halomonas* | 0.0 | 0.0 | 0.0 | 0.0 | 0.0 | 0.3 | 0.2 | 0.1 | 0.0 | 0.0 | 9.7 | 8.3 |

Supplement T 3: continued.

|  |  |  | **cDNA** | | | | | | | | | | | |
| --- | --- | --- | --- | --- | --- | --- | --- | --- | --- | --- | --- | --- | --- | --- |
|  |  |  | **Female** | | | | | **Male** | | | | | **Sea water** | |
| **Class** | **Family** | **Genus** | **7m** | **9m** | **10m** | **11m** | **12m** | **1m** | **2m** | **4m** | **5m** | **6m** | **RR1** | **RR2** |
| *Gammaproteobacteria* | *Methylomonaceae* | IheB2-23 | 12.6 | 5.6 | 5.6 | 5.5 | 1.4 | 1.9 | 4.9 | 0.8 | 1.5 | 11.7 | 0.0 | 0.0 |
| *Gammaproteobacteria* | *Methylomonaceae* | Marine Methylotrophic Group 2 | 2.2 | 2.5 | 2.8 | 1.9 | 0.3 | 3.0 | 4.2 | 4.8 | 5.4 | 6.9 | 0.2 | 0.3 |
| *Gammaproteobacteria* | *Methylophagaceae* | Marine Methylotrophic Group 3 | 0.3 | 0.6 | 0.4 | 0.2 | 0.3 | 0.2 | 0.5 | 0.0 | 0.6 | 0.8 | 0.0 | 0.0 |
| *Gammaproteobacteria* | *Methylomonaceae* | *Methyloprofundus* | 0.5 | 1.5 | 1.2 | 0.5 | 0.4 | 0.8 | 1.0 | 0.0 | 1.4 | 1.1 | 4.2 | 3.4 |
| *Gammaproteobacteria* | *Methylomonaceae* | Milano-WF1B-03 | 3.8 | 1.9 | 5.4 | 0.6 | 0.5 | 2.1 | 6.9 | 1.3 | 2.6 | 3.9 | 1.1 | 0.8 |
| *Gammaproteobacteria* | *Pasteurellaceae* | *Pasteurella* | 0.6 | 0.9 | 0.4 | 0.5 | 1.8 | 0.3 | 0.7 | 5.2 | 1.0 | 0.8 | 0.0 | 0.0 |
| *Gammaproteobacteria* | *Methylomonaceae* | pLW-20 | 0.5 | 1.1 | 1.2 | 0.6 | 0.0 | 1.0 | 1.3 | 0.7 | 4.9 | 1.9 | 0.0 | 0.0 |
| *Gammaproteobacteria* | Un. *Gammaproteobacteria* | - | 1.5 | 0.3 | 1.4 | 0.2 | 0.2 | 1.6 | 2.7 | 0.6 | 0.9 | 0.9 | 0.0 | 0.0 |
| *Gammaproteobacteria* | *Methylomonaceae* | - | 4.4 | 0.8 | 2.7 | 0.4 | 0.3 | 2.1 | 2.7 | 0.2 | 0.5 | 1.3 | 0.0 | 0.0 |
| *Gammaproteobacteria* | SS1-B-06-26 | - | 0.0 | 0.0 | 0.0 | 0.0 | 0.0 | 0.1 | 0.1 | 0.0 | 0.0 | 0.0 | 6.9 | 5.8 |
| *Gammaproteobacteria* | *Thiotrichaceae* | - | 0.2 | 0.7 | 0.7 | 0.6 | 0.5 | 2.1 | 1.7 | 1.1 | 2.6 | 1.8 | 0.0 | 0.0 |
| *Gammaproteobacteria* | Un. UBA10353 marine group | - | 1.4 | 0.4 | 1.2 | 0.2 | 0.2 | 1.9 | 2.7 | 0.2 | 0.6 | 0.9 | 0.4 | 0.2 |
| OM190 | Unknown OM190 | Unknown OM190 | 7.2 | 2.4 | 9.4 | 1.0 | 0.9 | 5.5 | 6.6 | 3.4 | 4.3 | 7.4 | 1.4 | 1.2 |
| *Parcubacteria* | Unknown *Ca. Kaiserbacteria* | - | 0.0 | 0.0 | 0.0 | 0.0 | 0.0 | 0.0 | 0.0 | 0.0 | 0.0 | 0.0 | 0.1 | 0.2 |
| *Planctomycetacia* | *Pirellulaceae* | *Bythopirellula* | 0.2 | 0.0 | 0.2 | 0.0 | 0.0 | 0.1 | 0.0 | 0.0 | 0.1 | 0.0 | 0.0 | 0.0 |
| *Planctomycetacia* | *Pirellulaceae* | Pir4 lineage | 0.0 | 0.1 | 0.2 | 0.0 | 0.0 | 0.1 | 0.0 | 0.0 | 0.1 | 0.0 | 0.0 | 0.0 |
| *Planctomycetacia* | *Rubinisphaeraceae* | *Planctomicrobium* | 0.5 | 0.4 | 0.3 | 0.0 | 0.1 | 0.2 | 0.5 | 0.0 | 0.2 | 0.0 | 0.0 | 0.0 |
| *Planctomycetacia* | *Rubinisphaeraceae* | - | 0.2 | 0.1 | 0.1 | 0.0 | 0.0 | 0.2 | 0.3 | 0.0 | 0.1 | 0.2 | 0.3 | 0.2 |
| *Rhodothermia* | *Rhodothermaceae* | - | 1.7 | 0.3 | 0.8 | 0.1 | 0.1 | 0.7 | 1.4 | 0.3 | 0.1 | 0.5 | 0.0 | 0.0 |
| *Thermoanaerobaculia* | *Thermoanaerobaculaceae* | Subgroup 10 | 2.7 | 0.1 | 4.3 | 0.1 | 0.0 | 0.9 | 2.7 | 0.1 | 0.1 | 1.0 | 0.0 | 0.0 |
| vadinHA49 | Unknown vadinHA49 | - | 0.4 | 0.2 | 0.9 | 0.1 | 0.0 | 0.4 | 1.8 | 0.1 | 0.0 | 0.8 | 0.0 | 0.0 |
| *Verrucomicrobiae* | *Verrucomicrobiales* DEV007 | - | 1.0 | 0.3 | 0.5 | 0.1 | 0.1 | 0.1 | 0.2 | 0.0 | 0.0 | 0.2 | 0.4 | 0.2 |
| Others | *-* | - | 26.7 | 18.1 | 28.1 | 4.4 | 10.8 | 26.1 | 22.5 | 9.8 | 28.8 | 17.9 | 46.9 | 48.2 |

Supplement T 4: Share (in %) of each taxonomic unit listed below on the DNA bacterial community found in sea water and *M. alvisca* carapace samples. These values are visualised in Figure 6. Taxa <0.5 % abundance are summarised in "others". cDNA - transcribed RNA; *Ca*. - *Candidatus*, Un. - unknown

|  |  |  | **DNA** | | | | | | | | | | | |
| --- | --- | --- | --- | --- | --- | --- | --- | --- | --- | --- | --- | --- | --- | --- |
|  |  |  | **Female** | | | | | **Male** | | | | | **Sea water** | |
| **Class** | **Family** | **Genus** | **7m** | **9m** | **10m** | **11m** | **12m** | **1m** | **2m** | **4m** | **5m** | **6m** | **RR1** | **RR2** |
| *Acidimicrobiia* | *Microtrichaceae* | Sva0996 marine group | 1.8 | 1.4 | 0.9 | 1.1 | 1.1 | 1.3 | 1.5 | 0.7 | 0.7 | 1.2 | 0.5 | 0.8 |
| *Acidimicrobiia* | Unknown *Actinomarinales* | - | 1.0 | 0.8 | 1.6 | 3.6 | 2.6 | 2.5 | 2.4 | 2.3 | 0.3 | 1.8 | 0.0 | 0.1 |
| *Actinobacteria* | *Nocardioidaceae* | *Nocardioides* | 0.0 | 0.0 | 0.0 | 0.0 | 0.0 | 0.0 | 0.0 | 0.0 | 0.0 | 0.0 | 5.5 | 3.8 |
| *Alphaproteobacteria* | *Rhodobacteraceae* | *Paracoccus* | 0.0 | 0.0 | 0.0 | 0.0 | 1.6 | 0.0 | 0.0 | 0.0 | 0.0 | 0.0 | 1.4 | 1.1 |
| *Alphaproteobacteria* | *Rhodobacteraceae* | *Sedimentitalea* | 0.1 | 0.2 | 0.5 | 0.2 | 0.7 | 0.3 | 0.3 | 0.9 | 0.5 | 0.5 | 0.0 | 0.0 |
| *Alphaproteobacteria* | *Rhodobacteraceae* |  | 1.2 | 1.2 | 2.5 | 2.7 | 6.8 | 2.1 | 1.3 | 2.0 | 1.5 | 2.2 | 5.2 | 3.3 |
| *Alphaproteobacteria* | *Sphingomonadaceae* |  | 0.0 | 0.0 | 0.0 | 0.0 | 0.0 | 0.0 | 0.0 | 0.0 | 0.0 | 0.0 | 8.9 | 9.1 |
| *Bacteroidia* | *Flavobacteriaceae* | *Formosa* | 1.1 | 1.2 | 2.9 | 0.9 | 3.2 | 2.5 | 0.6 | 1.7 | 1.2 | 0.8 | 0.0 | 0.0 |
| *Bacteroidia* | *Cyclobacteriaceae* | *Fulvivirga* | 0.4 | 0.4 | 0.3 | 0.7 | 1.2 | 1.3 | 1.9 | 1.4 | 0.6 | 1.3 | 0.0 | 0.0 |
| *Bacteroidia* | *Flavobacteriaceae* | *Lutimonas* | 1.6 | 1.2 | 1.7 | 1.6 | 2.8 | 2.8 | 1.5 | 2.2 | 0.4 | 1.1 | 0.0 | 0.0 |
| *Bacteroidia* | *Flavobacteriaceae* | *Mesonia* | 0.0 | 0.0 | 0.0 | 0.0 | 0.0 | 0.0 | 0.0 | 0.0 | 0.0 | 0.0 | 1.6 | 1.8 |
| *Bacteroidia* | *Saprospiraceae* | *Portibacter* | 1.1 | 1.2 | 1.1 | 0.2 | 0.5 | 1.0 | 0.4 | 1.2 | 0.9 | 0.7 | 0.0 | 0.0 |
| *Bacteroidia* | *Flavobacteriaceae* | *Ulvibacter* | 3.6 | 2.4 | 2.2 | 2.6 | 2.9 | 5.6 | 2.4 | 3.5 | 1.9 | 2.8 | 0.0 | 0.0 |
| *Bacteroidia* | *Cyclobacteriaceae* | - | 1.4 | 1.4 | 1.1 | 2.2 | 0.3 | 1.6 | 1.6 | 1.6 | 0.9 | 1.1 | 0.0 | 0.0 |
| *Bacteroidia* | *Flavobacteriaceae* | - | 8.3 | 4.3 | 5.7 | 8.2 | 14.2 | 6.7 | 4.5 | 6.4 | 3.4 | 5.1 | 1.2 | 0.8 |
| *Bacteroidia* | *Saprospiraceae* | - | 1.3 | 1.3 | 2.5 | 1.3 | 1.7 | 0.8 | 1.5 | 1.8 | 0.8 | 1.0 | 0.0 | 0.0 |
| *Deltaproteobacteria* | *Haliangiaceae* | *Haliangium* | 0.1 | 0.2 | 0.2 | 0.4 | 0.2 | 0.2 | 0.9 | 1.0 | 0.3 | 0.3 | 0.0 | 0.0 |
| *Deltaproteobacteria* | Unknown NB1-j | - | 0.6 | 0.3 | 0.5 | 0.3 | 0.3 | 0.4 | 0.5 | 0.4 | 0.4 | 0.1 | 0.3 | 0.4 |
| *Gammaproteobacteria* | *Arenicellaceae* | *Arenicella* | 1.0 | 0.7 | 1.1 | 0.8 | 2.2 | 1.3 | 1.5 | 1.2 | 0.9 | 1.0 | 0.0 | 0.0 |
| *Gammaproteobacteria* | *Beggiatoaceae* | *Ca.* *Marithrix* | 0.9 | 0.7 | 2.3 | 0.1 | 0.0 | 1.3 | 0.1 | 0.3 | 0.5 | 0.5 | 0.0 | 0.0 |
| *Gammaproteobacteria* | *Enterobacteriaceae* | *Escherichia*-*Shigella* | 0.0 | 0.0 | 0.0 | 0.0 | 0.0 | 0.0 | 0.0 | 0.0 | 0.0 | 0.0 | 0.0 | 0.0 |
| *Gammaproteobacteria* | *Halomonadaceae* | *Halomonas* | 0.0 | 0.0 | 0.0 | 0.0 | 0.0 | 0.0 | 0.0 | 0.0 | 0.0 | 0.0 | 6.0 | 5.0 |

Supplement T 4: continued.

|  |  |  | **DNA** | | | | | | | | | | | |
| --- | --- | --- | --- | --- | --- | --- | --- | --- | --- | --- | --- | --- | --- | --- |
|  |  |  | **Female** | | | | | **Male** | | | | | **Sea water** | |
| **Class** | **Family** | **Genus** | **7m** | **9m** | **10m** | **11m** | **12m** | **1m** | **2m** | **4m** | **5m** | **6m** | **RR1** | **RR2** |
| *Gammaproteobacteria* | *Methylomonaceae* | IheB2-23 | 4.0 | 1.2 | 1.7 | 3.9 | 0.3 | 1.1 | 1.9 | 0.9 | 0.5 | 2.9 | 0.0 | 0.0 |
| *Gammaproteobacteria* | *Methylomonaceae* | Marine Methylotrophic Group 2 | 5.0 | 7.8 | 6.6 | 11.4 | 1.9 | 10.4 | 10.4 | 18.7 | 14.7 | 18.9 | 0.0 | 0.1 |
| *Gammaproteobacteria* | *Methylophagaceae* | Marine Methylotrophic Group 3 | 0.3 | 0.6 | 1.6 | 1.9 | 4.5 | 0.8 | 0.8 | 1.7 | 2.8 | 1.8 | 0.0 | 0.0 |
| *Gammaproteobacteria* | *Methylomonaceae* | *Methyloprofundus* | 1.1 | 2.3 | 4.1 | 4.1 | 1.8 | 1.5 | 1.3 | 1.1 | 1.6 | 2.3 | 1.5 | 2.6 |
| *Gammaproteobacteria* | *Methylomonaceae* | Milano-WF1B-03 | 3.1 | 3.4 | 0.5 | 1.7 | 1.5 | 2.0 | 4.5 | 1.0 | 1.1 | 2.3 | 0.6 | 0.7 |
| *Gammaproteobacteria* | *Pasteurellaceae* | *Pasteurella* | 0.0 | 0.1 | 0.0 | 0.1 | 0.0 | 0.0 | 0.0 | 0.0 | 0.1 | 0.1 | 0.0 | 0.0 |
| *Gammaproteobacteria* | *Methylomonaceae* | pLW-20 | 1.7 | 1.7 | 3.6 | 2.5 | 0.0 | 2.1 | 1.4 | 2.6 | 5.6 | 1.7 | 0.0 | 0.0 |
| *Gammaproteobacteria* | Un. *Gammaproteobacteria* | - | 2.2 | 1.8 | 0.8 | 1.1 | 0.9 | 3.4 | 2.8 | 4.9 | 0.8 | 1.4 | 0.0 | 0.0 |
| *Gammaproteobacteria* | *Methylomonaceae* | - | 0.9 | 1.2 | 0.8 | 0.5 | 0.4 | 0.4 | 0.6 | 0.0 | 0.4 | 0.6 | 0.0 | 0.0 |
| *Gammaproteobacteria* | SS1-B-06-26 | - | 0.0 | 0.0 | 0.0 | 0.0 | 0.0 | 0.0 | 0.0 | 0.0 | 0.0 | 0.0 | 1.2 | 1.7 |
| *Gammaproteobacteria* | *Thiotrichaceae* | - | 0.3 | 0.2 | 0.9 | 1.9 | 1.0 | 0.8 | 0.6 | 0.8 | 0.8 | 1.0 | 0.0 | 0.0 |
| *Gammaproteobacteria* | Un.UBA10353 marine group | - | 2.0 | 1.2 | 2.1 | 2.7 | 4.7 | 2.8 | 2.1 | 1.8 | 1.5 | 1.7 | 0.6 | 0.6 |
| OM190 | Unknown OM190 | Unknown OM190 | 5.3 | 6.4 | 4.7 | 2.8 | 1.0 | 5.2 | 5.3 | 4.8 | 6.9 | 7.2 | 2.0 | 2.8 |
| *Parcubacteria* | Unknown *Ca. Kaiserbacteria* | - | 1.4 | 0.3 | 1.5 | 0.8 | 2.0 | 0.9 | 1.2 | 1.0 | 1.6 | 1.1 | 1.3 | 1.5 |
| *Planctomycetacia* | *Pirellulaceae* | *Bythopirellula* | 2.5 | 5.4 | 1.8 | 1.9 | 0.2 | 1.9 | 2.0 | 0.8 | 2.4 | 2.0 | 0.0 | 0.0 |
| *Planctomycetacia* | *Pirellulaceae* | Pir4 lineage | 1.4 | 3.3 | 3.9 | 2.9 | 0.8 | 1.2 | 1.3 | 1.2 | 4.5 | 1.6 | 0.2 | 0.2 |
| *Planctomycetacia* | *Rubinisphaeraceae* | *Planctomicrobium* | 5.4 | 7.8 | 6.5 | 4.2 | 1.3 | 3.1 | 3.4 | 2.4 | 7.6 | 3.1 | 0.0 | 0.0 |
| *Planctomycetacia* | *Rubinisphaeraceae* | - | 0.8 | 1.4 | 0.3 | 0.0 | 0.0 | 0.8 | 0.8 | 0.6 | 0.5 | 0.6 | 14.3 | 11.9 |
| *Rhodothermia* | *Rhodothermaceae* | - | 0.7 | 0.7 | 0.4 | 0.5 | 0.2 | 1.1 | 1.1 | 0.6 | 0.2 | 0.4 | 0.0 | 0.0 |
| *Thermoanaerobaculia* | *Thermoanaerobaculaceae* | Subgroup 10 | 1.3 | 1.1 | 0.1 | 0.6 | 0.3 | 0.9 | 2.3 | 0.2 | 0.1 | 0.6 | 0.0 | 0.0 |
| vadinHA49 | Unknown vadinHA49 | - | 0.7 | 1.8 | 0.6 | 1.9 | 0.5 | 1.2 | 2.4 | 1.1 | 0.3 | 2.2 | 0.0 | 0.0 |
| *Verrucomicrobiae* | *Verrucomicrobiales* DEV007 | - | 2.3 | 1.6 | 1.2 | 0.9 | 0.8 | 1.0 | 2.1 | 0.7 | 0.2 | 0.9 | 0.6 | 0.6 |
| Others | *-* | - | 32.1 | 30.0 | 28.8 | 24.9 | 33.2 | 25.9 | 29.0 | 24.7 | 30.7 | 23.9 | 46.8 | 51.2 |

Supplement T 5: Share (in %) of each taxonomic unit listed below on the total archaeal community found in sea water and *M. alvisca* carapace samples. These values are visualised in Figure 7. Taxa <0.5 % abundance are summarised in "others". cDNA - transcribed RNA, *Ca*. - *Candidatus*

|  |  |  | **cDNA** | | | | | | | | | | | |
| --- | --- | --- | --- | --- | --- | --- | --- | --- | --- | --- | --- | --- | --- | --- |
|  |  |  | **Female** | | | | | **Male** | | | | | **Sea water** | |
| **Class** | **Family** | **Genus** | **7m** | **9m** | **10m** | **11m** | **12m** | **1m** | **2m** | **4m** | **5m** | **6m** | **RR1** | **RR2** |
| *Nitrososphaeria* | *Nitrosopumilaceae* | *Ca.* *Nitrosopumilus* | 100.0 | 100.0 | 100.0 | 100.0 | 100.0 | 99.3 | 99.8 | 99.7 | 100.0 | 99.4 | 67.9 | 66.0 |
| *Nitrososphaeria* | *Nitrosopumilaceae* | - | 0.0 | 0.0 | 0.0 | 0.0 | 0.0 | 0.0 | 0.0 | 0.3 | 0.0 | 0.0 | 1.9 | 0.9 |
| *Thermoplasmata* | Unknown Marine Group II | - | 0.0 | 0.0 | 0.0 | 0.0 | 0.0 | 0.0 | 0.0 | 0.0 | 0.0 | 0.0 | 25.3 | 27.5 |
| *Thermoplasmata* | Marine Group III | - | 0.0 | 0.0 | 0.0 | 0.0 | 0.0 | 0.0 | 0.0 | 0.0 | 0.0 | 0.0 | 3.5 | 2.7 |
| *Woesearchaeia* | - | - | 0.0 | 0.0 | 0.0 | 0.0 | 0.0 | 0.7 | 0.2 | 0.0 | 0.0 | 0.6 | 0.7 | 1.4 |
| Others | - | - | 0.0 | 0.0 | 0.0 | 0.0 | 0.0 | 0.0 | 0.0 | 0.0 | 0.0 | 0.0 | 0.8 | 1.5 |
|  |  |  |  |  |  |  |  |  |  |  |  |  |  |  |
|  |  |  | **DNA** | | | | | | | | | | | |
|  |  |  | **Female** | | | | | **Male** | | | | | **Sea water** | |
| **Class** | **Family** | **Genus** | **7m** | **9m** | **10m** | **11m** | **12m** | **1m** | **2m** | **4m** | **5m** | **6m** | **RR1** | **RR2** |
| *Nitrososphaeria* | *Nitrosopumilaceae* | *Ca.* *Nitrosopumilus* | 98.5 | 99.2 | 95.7 | 99.2 | 97.2 | 98.5 | 99.2 | 99.2 | 91.7 | 98.2 | 55.2 | 59.4 |
| *Nitrososphaeria* | *Nitrosopumilaceae* | - | 0.0 | 0.0 | 0.2 | 0.0 | 0.0 | 0.0 | 0.0 | 0.1 | 0.9 | 0.0 | 9.4 | 9.5 |
| *Thermoplasmata* | Unknown Marine Group II | - | 0.1 | 0.0 | 0.4 | 0.0 | 1.0 | 0.1 | 0.0 | 0.1 | 2.2 | 0.1 | 23.3 | 23.7 |
| *Thermoplasmata* | Marine Group III | - | 0.0 | 0.0 | 0.2 | 0.0 | 0.0 | 0.0 | 0.0 | 0.0 | 0.0 | 0.0 | 4.9 | 5.2 |
| *Woesearchaeia* | - | - | 1.1 | 0.6 | 1.9 | 0.5 | 0.6 | 1.3 | 0.5 | 0.0 | 2.0 | 1.4 | 3.0 | 0.9 |
| Others | - | - | 0.3 | 0.1 | 1.7 | 0.4 | 1.2 | 0.1 | 0.2 | 0.6 | 3.2 | 0.3 | 4.2 | 1.3 |

## Microbial ASVs used in different analysis

Supplement T 6: Most abundant (as defined in the methods section) archaeal amplicon sequence variants (aASVs) used in the display of microdiversity via heatmaps (Figure 5A) and for construction of phylogenetic trees (Supplement F 1) including unique identifier of sequence. *Sequence not detected in epibiotic datasets, but in sea water samples, therefore included in analysis.

| **ID** | **Identifier** | **Affiliation** | **Share, DNA [%]** | | **Share, RNA [%]** | |
| --- | --- | --- | --- | --- | --- | --- |
|  |  |  | **Male** | **Female** | **Male** | **Female** |
| aASV_1 | 256f833880283d08ef8165da4e44fcbd | *Candidatus Nitrosopumilus* | 25.09 | 15.07 | 0 | 0 |
| aASV_2 | 57c754d3fe89d0c0c85b6e84a9b3cf32 | *Candidatus Nitrosopumilus* | 9.18 | 6.56 | 0 | 0 |
| aASV_3 | 631cea89230e804ad9ee38afda885416 | *Candidatus Nitrosopumilus* | 13.92 | 17.78 | 0 | 0 |
| aASV_4 | cdda5caad60a3fc33af2c95c5c228edb | *Candidatus Nitrosopumilus* | 27.24 | 31.29 | 0 | 0 |
| aASV_5 | d4922b1aa24b5cf063125b67eea01001 | *Candidatus Nitrosopumilus* | 5.75 | 6.01 | 0 | 0 |
| aASV_6 | e291b780b2c88c2fecd1992280ea012d | *Candidatus Nitrosopumilus* | 0.74 | 0.79 | 0 | 0 |
| aASV_7 | f196686853cbece5d6b9f00a8b1365fa | *Candidatus Nitrosopumilus* | 7.13 | 8.93 | 0 | 0 |
| aASV_8 | f9252dfa4e5bc371c27f99b00629de20 | *Candidatus Nitrosopumilus* | 1.60 | 0.33 | 0 | 0 |
| aASV_9 | 00b6ba450d8fa0d7b5f170a21bab985e | *Candidatus Nitrosopumilus* | 0.17 | 0 | 0 | 0 |
| aASV_10 | 02956adbcc9981e618587e8d7f9a71fe | *Candidatus Nitrosopumilus* | 0.08 | 0.10 | 0 | 0.08 |
| aASV_11 | 0ae5df471c6243574b958d7d60680906 | *Candidatus Nitrosopumilus* | 0.23 | 0 | 0.58 | 0 |
| aASV_12 | 156adfd009d6b4e39b726068f009ddef | *Candidatus Nitrosopumilus* | 0.27 | 0 | 0.05 | 0 |
| aASV_13* | 1d4ad07c4256172a27d6abf9656dcae1 | *Candidatus Nitrosopumilus* | 0 | 0 | 0 | 0 |
| aASV_14 | 2254b92ebe4394541a526d85f5996ff4 | *Candidatus Nitrosopumilus* | 0.19 | 0.05 | 0 | 0 |
| aASV_15 | 2a50957ab839fe90ad36e6a06d6c1ff5 | *Candidatus Nitrosopumilus* | 0 | 1.34 | 0 | 0 |
| aASV_16* | 2babe199eeb96a80fa195203d5febb24 | *Candidatus Nitrosopumilus* | 0 | 0 | 0 | 0 |
| aASV_17 | 30af0d5887e569ec4d6389c5b451c999 | *Candidatus Nitrosopumilus* | 0.19 | 0 | 0 | 0 |
| aASV_18 | 3fe8c53cb965cf4991ba7893a58dbaae | *Candidatus Nitrosopumilus* | 0.02 | 0.19 | 0 | 0 |
| aASV_19 | 449114fe4cdc98a42158f8404de815e9 | *Candidatus Nitrosopumilus* | 0 | 0.14 | 0 | 0 |
| aASV_20 | 47b796758dc3a86e7d9f3a0ef4d39d78 | *Candidatus Nitrosopumilus* | 1.04 | 0.42 | 0.44 | 0 |
| aASV_21 | 4915112495993a1aa6be626b002a9012 | *Candidatus Nitrosopumilus* | 0.13 | 0.29 | 0 | 0.53 |
| aASV_22 | 4be87b47b466833997b8a16ed5199858 | *Candidatus Nitrosopumilus* | 0 | 0.14 | 0 | 0 |
| aASV_23 | 52953189e1e1e0f059c2ebb0ef4c179a | *Candidatus Nitrosopumilus* | 0 | 0 | 0.09 | 0 |
| aASV_24 | 52cfe745088a6dca4371ad332a5fb691 | *Candidatus Nitrosopumilus* | 0.31 | 0 | 0 | 0 |
| aASV_25 | 588387b64686dfcb09eda9a70006129c | *Candidatus Nitrosopumilus* | 0 | 0 | 0 | 0.76 |
| aASV_26 | 603d3672085bd1878bdccd70200c572e | *Candidatus Nitrosopumilus* | 0 | 0 | 0.07 | 0 |
| aASV_27 | 6cffe81394e2e32935f3f91bbd2273de | *Candidatus Nitrosopumilus* | 0 | 0 | 0.38 | 0 |
| aASV_28 | 7289512799ed2c3688521dda2b5fafed | *Candidatus Nitrosopumilus* | 2.44 | 4.58 | 0.19 | 0 |
| aASV_29 | 7289b5b21fe8126481b2243ce8768c2e | *Candidatus Nitrosopumilus* | 0 | 0 | 0.42 | 0 |
| aASV_30* | 783497cb5c48d2e46feb2eaa09a0159c | *Candidatus Nitrosopumilus* | 0 | 0 | 0 | 0 |
| aASV_31 | 78df39e56c38b30a20d4315de09faa66 | *Candidatus Nitrosopumilus* | 0.16 | 0.05 | 0.10 | 0.05 |
| aASV_32 | 7c39e222911b925f36735e662cd2d133 | *Candidatus Nitrosopumilus* | 0 | 0 | 0.32 | 0 |
| aASV_33 | 823ae71fdb7652e80db103858a8b3e9e | *Candidatus Nitrosopumilus* | 0 | 0.36 | 0 | 0 |
| aASV_34 | 82626e91bcd71fe63d5545a4a2fea4ec | *Candidatus Nitrosopumilus* | 0 | 0 | 0.34 | 0 |
| aASV_35 | 853c32c816e7c3805e4a8c671133174e | *Candidatus Nitrosopumilus* | 0.03 | 0.14 | 0.01 | 0 |
| aASV_36 | 9c13ab1481425515a1cb7d648ac12b38 | *Candidatus Nitrosopumilus* | 0 | 0 | 0 | 0.40 |
| aASV_37 | 9d8d2f40465e9477f8c95dfd17aca24b | *Candidatus Nitrosopumilus* | 0.43 | 0.61 | 0.64 | 0.43 |
| aASV_38 | 9e392f88ea28d7b919c0d496094e47a8 | *Candidatus Nitrosopumilus* | 0 | 0.31 | 0 | 0 |
| aASV_39 | a87120bfc38a0fd1e964993709d6026d | *Candidatus Nitrosopumilus* | 0.01 | 0.06 | 0 | 0 |
| aASV_40* | aa317854251df4ab0aa5e87f6fc08e14 | *Candidatus Nitrosopumilus* | 0 | 0 | 0 | 0 |
| aASV_41 | ae164f42fac1a0bac3a53947efc447b3 | *Candidatus Nitrosopumilus* | 0.06 | 0.45 | 0 | 0 |
| aASV_42 | bab94556cff8d3e80faaa740c230d49e | *Candidatus Nitrosopumilus* | 0 | 0.56 | 0 | 1.19 |
| **Supplement T 6:** continued. | | | | | | |
| **ID** | **Identifier** | **Affiliation** | **Share, DNA [%]** | | **Share, RNA [%]** | |
|  |  |  | **Male** | **Female** | **Male** | **Female** |
| aASV_43 | bbd5f1516d05e30a9c283f9c41eeaa5f | *Candidatus Nitrosopumilus* | 0.15 | 0 | 0 | 0 |
| aASV_44 | bc48a282733f7e11866aa84e0d10dc29 | *Candidatus Nitrosopumilus* | 0 | 0.12 | 0.38 | 0 |
| aASV_45 | e05aeba6407701e0b64f713d6f3eefd1 | *Candidatus Nitrosopumilus* | 0.11 | 0.24 | 0.02 | 0 |
| aASV_46 | e36222b0b23b48e88830986b821546f1 | *Candidatus Nitrosopumilus* | 0 | 0 | 0.17 | 0 |
| aASV_47 | e66a1c7a8dc053f7ec7c389b7adac7a4 | *Candidatus Nitrosopumilus* | 0.13 | 0 | 0 | 0 |
| aASV_48* | e6e122c4a09e361870d703d892e150ae | *Candidatus Nitrosopumilus* | 0 | 0 | 0 | 0 |
| aASV_49 | ec5fe78009943ba8b63c600b4871532e | *Candidatus Nitrosopumilus* | 0 | 0 | 0.25 | 0 |
| aASV_50 | fa82f8d64c3d8dc799b028759eb1a20b | *Candidatus Nitrosopumilus* | 0.13 | 0 | 0 | 0 |
| aASV_51 | fa846f87bd6d58be5ac200fb50903ed1 | *Candidatus Nitrosopumilus* | 0 | 0.27 | 0 | 0 |
| aASV_52 | fc133c54591919ee5893cf2fd86b6d31 | *Candidatus Nitrosopumilus* | 0.14 | 0.32 | 0 | 0.12 |
| aASV_53 | fc73d2bbdd1cf68499d69da4e4e7743f | *Candidatus Nitrosopumilus* | 0.11 | 0 | 0 | 0 |
| aASV_54* | fe2449648b43ca517a79bb7f6ec27792 | *Candidatus Nitrosopumilus* | 0 | 0 | 0 | 0 |

Supplement T 7: Most abundant (as defined in the methods section) bacterial amplicon sequence variants (bASVs) used in the display of microdiversity via heatmaps (Figure 5A) and for construction of phylogenetic trees (Supplement F 2) including unique identifier of sequence. *Sequence not detected in epibiotic datasets, but in sea water samples, therefore included in analysis.

| **ID** | **Identifier** | **Affiliation** | **Share, DNA [%]** | | **Share, RNA [%]** | |
| --- | --- | --- | --- | --- | --- | --- |
|  |  |  | **Male** | **Female** | **Male** | **Female** |
| bASV_1 | 0152e83b43a2b9f518860ed2bfcdc084 | Marine Methylotrophic Group 2 | 1.70 | 0.17 | 0 | 0 |
| bASV_3 | 02d585b47208f06e50c8b4d2a116f4a6 | *Methyloprofundus* sp. | 0.76 | 1.11 | 0 | 0 |
| bASV_14 | 186e34c7b060bb9789913d9711ebb7ca | *Planctomicrobium* sp. | 2.27 | 3.19 | 0 | 0 |
| bASV_19 | 2615744b4ddd3b252bd00a94a2ed6b1e | Unknown *Flavobacteriaceae* | 2.13 | 4.17 | 0.32 | 0.64 |
| bASV_27 | 3f86bae5a8794f60f41d307d1b69854f | Unknown *Cyclobacteriaceae* | 0.97 | 0.89 | 0.35 | 0.24 |
| bASV_29 | 445adc5125dc9c9a96c0837e007449d3 | Unknown *Flavobacteriaceae* | 0.86 | 1.09 | 0 | 0 |
| bASV_30 | 46a16ed0a98a23cd9886f6758b2c42d3 | *Fulvivirga* sp. | 0.98 | 0.44 | 0 | 0 |
| bASV_38 | 5ae89b41a62300dcc6fd9b0b4eaa2608 | Marine Methylotrophic Group 2 | 3.10 | 0.37 | 0 | 0 |
| bASV_45 | 67b091be2ff03a7eef7a842f5f68d756 | Pir4 (*Pirellulaceae*) | 1.00 | 1.16 | 0 | 0 |
| bASV_58 | 7c36ab11160b7a75ea555a28d549b4e0 | *Formosa* sp. | 0.80 | 0.98 | 0 | 0 |
| bASV_59 | 8505d73015fcd4aff97e115ef407dd9d | Marine Methylotrophic Group 2 | 2.19 | 2.04 | 0 | 0 |
| bASV_69 | 96c7be9672f5dd690cd0f2f4a5529853 | IheB2-23 (Methylomonaceae) | 0.39 | 0.92 | 2.23 | 2.85 |
| bASV_79 | aa570a98342daed3171333dd474aefa4 | *Planctomicrobium* sp. | 0.48 | 1.03 | 0 | 0 |
| bASV_83 | b2cf616c1f58ae72953e55e464cafc03 | Unknown *Actinomarinales* | 1.75 | 1.90 | 0.40 | 0.21 |
| bASV_84 | b98860d17c77371c19aee1d6bd0bafa2 | *Ulvibacter* sp. | 1.61 | 1.76 | 0 | 0 |
| bASV_102 | d86891ad5baa3060689415f0e1e50862 | vadinHA49 (*Planctomycetes*) | 1.19 | 0.87 | 0.13 | 0.22 |
| bASV_105 | e14e43b644b76ff2eab0649d28cfe295 | *Methylomonaceae* | 0.17 | 0.29 | 1.29 | 1.52 |
| bASV_107 | e82f3515d453987eaff124ef0cb4576c | *Candidatus Marithrix* | 0.53 | 0.83 | 10.45 | 4.23 |
| bASV_110 | f47ee451b3c6fc97d6277824c1183319 | Unknown *Gammaproteobacteria* | 0.97 | 0.84 | 0 | 0 |
| bASV_112 | fc38e3f66f6feab6b39182561e881809 | *Sedimentitalea* sp. | 0.44 | 0.23 | 2.59 | 0.42 |
| bASV_113 | fc4eef576bc07d7885d263092e130fe8 | pLW-20 (*Methylomonaceae*) | 2.67 | 1.88 | 1.91 | 0.67 |
| bASV_118 | 47311034d26872902e151c2c7a070256 | *Escherichia-Shigella* sp. | 0 | 0 | 2.90 | 3.85 |
| bASV_119 | 6d0a842273291c1078e3c1fa5d629067 | *Pasteurella* sp. | 0 | 0 | 1.60 | 0.81 |
| bASV_123 | ffc36e27c82042664a16bcd4d380b286 | *Escherichia-Shigella* sp. | 0 | 0 | 22.25 | 29.43 |
| bASV_124* | 0564b7b141449cb62970baed1e86651d | *Albimonas* *pacifica* | 0 | 0 | 0 | 0 |
| bASV_125 | 0be920ddc5904f0e4c2a1cfc21f5bc34 | Unknown *Rhodobacteraceae* | 0 | 0 | 0 | 0 |
| bASV_126 | 17296c6371409d21052021cfdf74feea | Unknown *Rhodobacteraceae* | 0.09 | 0.23 | 0.05 | 0.13 |
| bASV_127 | 1fc889d19966a9dda4fd0efb7600aac8 | Roseobacter clade NAC11-7 lineage | 0.09 | 0.04 | 0.05 | 0.02 |
| bASV_128 | 26a6b3a04407a589d9695f81e56886c4 | *Yangia* sp. | 0 | 0 | 0.01 | 0 |
| bASV_129* | 272fc0c0fc6df72dcab0bd8feaeb01bb | *Sulfitobacter* sp. | 0 | 0 | 0 | 0 |
| bASV_130 | 2d9c3b4de017ef3a0e576ce068dd8de8 | *Paracoccus* sp. | 0 | 0.26 | 0 | 0.04 |
| bASV_131 | 2fc9a3f8b79271fe15af624fec96322d | *Thioclava* sp. | 0 | 0 | 0.01 | 0 |
| bASV_132* | 372a73475e6b28ec68754dc59e88bdc1 | *Sulfitobacter* sp. | 0 | 0 | 0 | 0 |
| bASV_133 | 375981c9c5d7289c259139e4d9498e56 | Unknown *Rhodobacteraceae* | 0 | 0 | 0 | 0.09 |
| bASV_134 | 38c0d00b192d5fe812a53c31624ef6ad | Roseobacter clade NAC11-7 lineage | 0.04 | 0.04 | 0 | 0 |
| bASV_135 | 43dc52025c9097904b4053f599d47f0c | *Sedimentitalea* sp. | 0 | 0 | 0.04 | 0.01 |
| bASV_136 | 44030a9dc571556725b43b21c7ac866f | Unknown *Rhodobacteraceae* | 0 | 0 | 0 | 0 |
| bASV_137 | 4c9a4d5fec3b46eb387de1a4fa8ab7a1 | Unknown *Rhodobacteraceae* | 0.22 | 0.20 | 0.04 | 0.06 |
| bASV_138 | 53e426b05f2d7eb11fdcd959b1420d0b | *Roseobacter* clade NAC11-7 lineage | 0.06 | 0.14 | 0.01 | 0 |

| **Supplement T 7:** continued. | | | | | | |
| --- | --- | --- | --- | --- | --- | --- |
| **ID** | **Identifier** | **Affiliation** | **Share, DNA [%]** | | **Share, RNA [%]** | |
|  |  |  | **Male** | **Female** | **Male** | **Female** |
| bASV_139 | 60e03ba11ed28e9181481f51f6c88ff3 | *Pseudooceanicola* sp. | 0 | 0 | 0.02 | 0 |
| bASV_140* | 621a6e8ffefb0b9a6d23438065317218 | *Paracoccus* sp. | 0 | 0 | 0 | 0 |
| bASV_141 | 630860d80a9dc35ed55113eeb8a3e761 | *Paracoccus* sp. | 0 | 0 | 0.15 | 12.66 |
| bASV_142 | 6829983e928caf5aaa000d63a0600e5b | *Paracoccus* sp. | 0 | 0 | 0 | 0.19 |
| bASV_143 | 77000fa95996619079ef61d19bab8608 | Roseobacter clade NAC11-7 lineage | 0.25 | 0 | 0.09 | 0 |
| bASV_144 | 786416226f74a1d41f6db5d3c011f8be | *Sedimentitalea* sp. | 0 | 0.01 | 0 | 0.01 |
| bASV_145 | 7b4b32d773e629c1534fc70a81b53fb0 | Roseobacter clade NAC11-7 lineage | 0.17 | 0.14 | 0.03 | 0.03 |
| bASV_146 | 865ce03f2551309692371bfb60186a65 | Unknown *Rhodobacteraceae* | 0 | 0.03 | 0 | 0 |
| bASV_147 | 8d2bf2ad3d4b7a9eb0673e70208f0b33 | Unknown *Rhodobacteraceae* | 0.06 | 0.05 | 0.01 | 0.03 |
| bASV_148 | 8e37b72241df016ad11f97bbc5e87a23 | *Amaricoccus* sp. | 0 | 0 | 0.05 | 0 |
| bASV_149 | 9785298ff4200d459467b68dccbbbdea | *Sulfitobacter* sp. | 0 | 0 | 0.05 | 0 |
| bASV_150 | 97a98800430c042002c16c6ea4ef7ecd | *Amaricoccus* sp. | 0 | 0 | 0 | 0.02 |
| bASV_151* | 9aba136186b1db322b4ef52c5935cc45 | Unknown *Rhodobacteraceae* | 0 | 0 | 0 | 0 |
| bASV_152 | 9eabaf1c220792e04c32e85ee6a6c6ec | Unknown *Rhodobacteraceae* | 0.15 | 0.08 | 0.03 | 0.04 |
| bASV_153 | 9f811faf63df436c90c22610ff9e9307 | Unknown *Rhodobacteraceae* | 0 | 0 | 0.02 | 0 |
| bASV_154* | a7f3bafca8571b87c33c8495d2636db3 | Unknown *Rhodobacteraceae* | 0 | 0 | 0 | 0 |
| bASV_155 | c60dcbf34dee9e9c10c22659c9e676d2 | Unknown *Rhodobacteraceae* | 0 | 0.04 | 0 | 0 |
| bASV_156 | c7f263bb956d149bfda82ef7d85a8f45 | Unknown *Rhodobacteraceae* | 0.03 | 0.15 | 0 | 0 |
| bASV_157 | ccc1f684dc49332ca3e269eecb1a8948 | Unknown *Rhodobacteraceae* | 0.03 | 0.02 | 0.01 | 0 |
| bASV_158 | cf61de9ed6d0fe3cae13ddef7f56cc68 | Unknown *Rhodobacteraceae* | 0.09 | 0.19 | 0 | 0 |
| bASV_159 | d134aedb0886439ef47ca358aae1f242 | Roseobacter clade NAC11-7 lineage | 0.02 | 0.02 | 0.02 | 0 |
| bASV_160 | d17389d7933b755f5b4bcecece388657 | *Sedimentitalea* sp. | 0.05 | 0.10 | 0.02 | 0.13 |
| bASV_161 | d5efaef1c67fe453cfa5d6d2f8a68042 | *Paracoccus* sp. | 0 | 0 | 0.01 | 0.02 |
| bASV_162 | d9f1b33889f66de11ac2dca664a4f916 | Roseobacter clade NAC11-7 lineage | 0 | 0.03 | 0 | 0 |
| bASV_163 | da686b27b96522eb99b12ab2ac370c3b | Unknown *Rhodobacteraceae* | 0.02 | 0.01 | 0.01 | 0 |
| bASV_164 | deaf8fa90c6ab86cee8d4c1bb3bd53ee | *Sulfitobacter* sp. | 0 | 0 | 0.01 | 0 |
| bASV_165 | e25096b0cf6cf0d7fca58b1564b39d85 | Unknown *Rhodobacteraceae* | 0 | 0 | 0.01 | 0 |
| bASV_166 | e696da7b21de6c846082ac52dbeb5dea | Unknown *Rhodobacteraceae* | 0.28 | 0.41 | 0.04 | 0.01 |
| bASV_167 | f08d9591c33dcd47c7c56c08b92a10e9 | Unknown *Rhodobacteraceae* | 0.04 | 0.01 | 0 | 0.01 |
| bASV_168 | f94d17d109e2a3785c86c384d87df418 | *Paracoccus* sp. | 0 | 0.04 | 0 | 0.01 |

## Phylogenetic analysis of most abundant epibiotic aASVs and bASVs


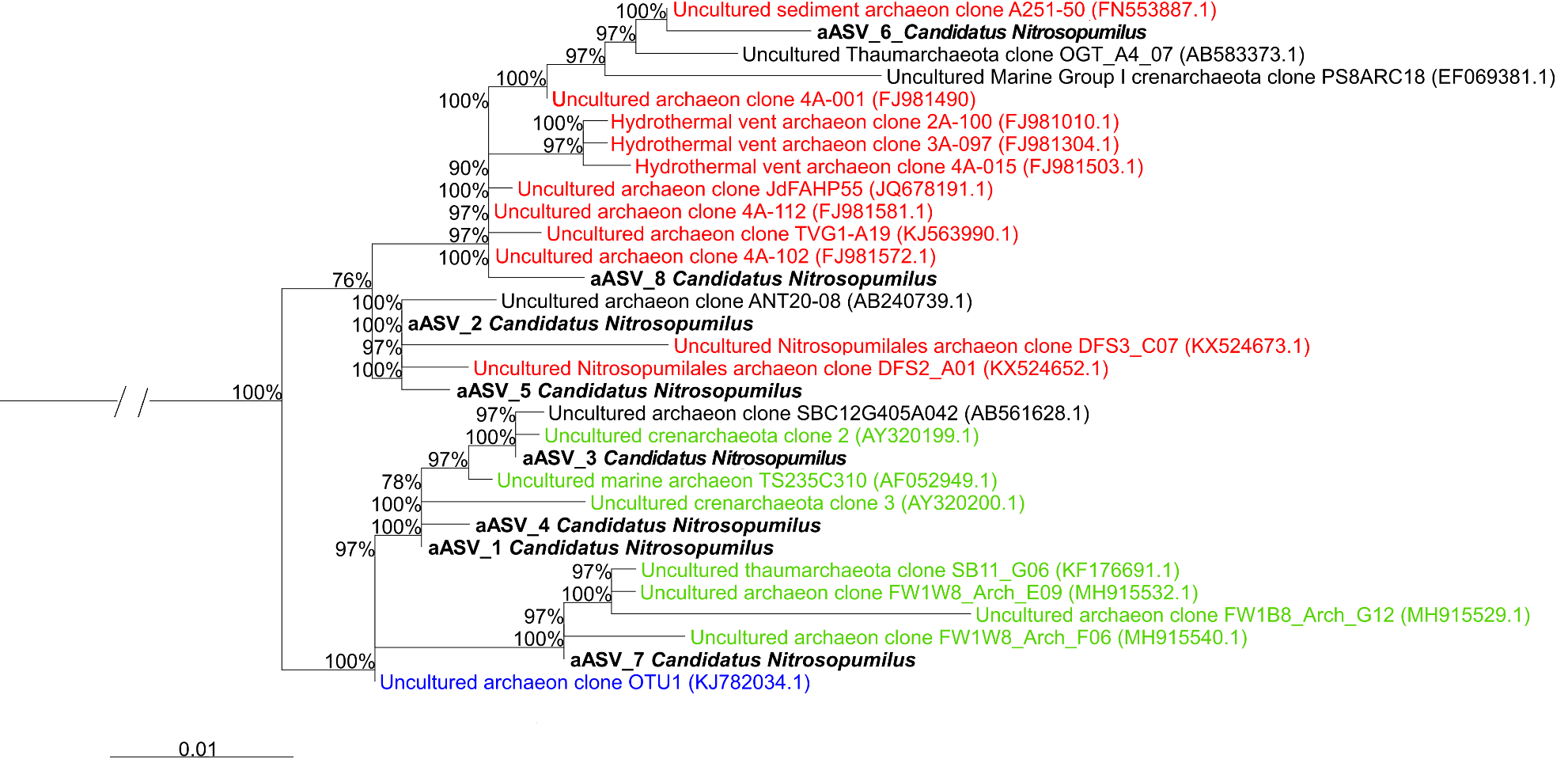
Those ASVs that were present in ≥70 % of all *Munidopsis alvisca* samples within one data set (i.e. *Archaea*, *Bacteria*) and that represent ≥0.95 % of the attached community were considered the “most abundant” representatives and were used for the phylogenetic analysis. Colour code and abbreviations in Supplement F 1 and Supplement F 2: green: association with host organisms; red: hydrothermal vent; blue: habitat enriched in hydrocarbons, asphalt, diesel or characterised by oil seep or spill.

Supplement F 1: Neighbor-joining tree of the most abundant epibiotic aASVs (≥0.95 %) in the combined RNA and DNA datasets and their closest relatives (identified by nBLAST search in the NCBI database). Only bootstrap values ≥50% (derived from 1,000 replicates) at main nodes are shown. Selected sequences related to *Thaumarchaeota* in the SILVA database were used as outgroup to define the root of the tree. Root not shown (cut at//). GenBank accession numbers are given in parentheses and abundances of aASVs are listed in Supplement T 4.


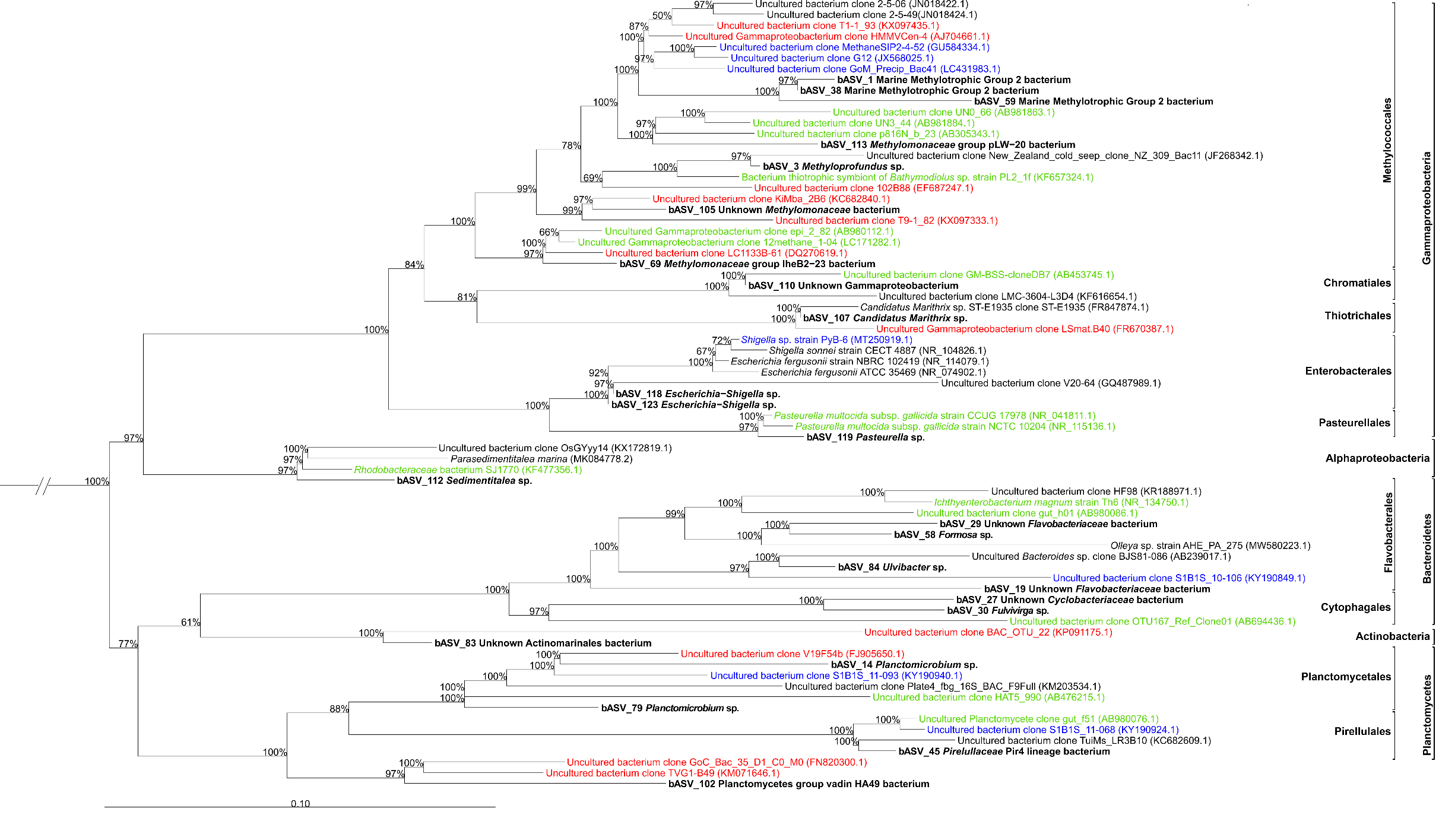


Supplement F 2: Neighbor-joining tree of the most abundant epibiotic bASVs (≥0.95 %) in the combined RNA and DNA datasets and their closest relatives (identified by nBLAST search in the NCBI database). Only bootstrap values ≥50% (derived from 1,000 replicates) at main nodes are shown. Selected sequences related to *Cyanobacteria* in the SILVA database were used as outgroup to define the root of the tree. Root not shown (cut at//). GenBank accession numbers are given in parentheses and abundances of bASVs are listed in Supplement T 3.

## Extraction of microbial nucleic acids

Samples were thawed on ice and treated with proteinase K (20 mg/ml, 37 °C, 30 min). Then, they were mixed with extraction buffer (50 mM sodium acetate, 10 mM EDTA, pH 4.2) and cells were disrupted by combined treatment with phenol-chloroform-isoamyl alcohol (ROTI-Aqua-P/C/I, Carl Roth, Karlsruhe, Germany) and bead beating (20 min). After centrifugation (10,000 rpm, 4 °C, 20 min), an upper aqueous phase (containing RNA) and a lower organic phase (containing DNA) had formed. The aqueous RNA-containing phase was collected in a fresh vessel and stored on ice. Following a second extraction step, which was included to increase the yield, both aqueous phases were combined. Remaining chemicals were removed from the RNA extract by washing with a chloroform-isoamyl alcohol solution (ROTI C/I, Carl Roth, Karlsruhe, Germany). The resulting aqueous phase was mixed with glycogen and RNA was precipitated at -20 °C overnight in the presence of 100 % isopropanol and 3 M sodium acetate (pH 4.8). The precipitate was washed with ice-cold ethanol (80 %), air-dried and re-dissolved in buffer.

DNA was extracted from the organic phase of the extraction mix with 1 M Tris-base buffer (pH 10.5) and subsequent centrifugation (5,200 rpm, 4 °C, 15 min). Extracted DNA was purified and processed as described for RNA but precipitated in 100 % ice-cold ethanol (modified protocol after [1]).

## Amplification of target genes for identification of organisms

### Eukarya

Supplement T 8: PCR settings and composition of reaction mix used for amplification of the mitochondrial cytochrome c oxidase subunit I (COI).

| **Reagent** | **Volume**  **[µl]** |  | **Cycles** | **Step** | **Temperature**  **[°C]** | **Time**  **[min]** |
| --- | --- | --- | --- | --- | --- | --- |
| AccuStart PCR SuperMix* | 12.5 |  | 1 | Initial denaturation | 94 | 03:00 |
| dd H_2_O | 9.5 |  |  |  |  |  |
| Primer jgLCO1490 (10 pmol) | 0.5 |  | 35 | Denaturation | 94 | 00:30 |
| Primer jgHCO2198 (10 pmol) | 0.5 |  |  | Primer annealing | 45 | 00:45 |
| Template | 2 |  |  | Elongation | 72 | 01:00 |
| Sum | 25 |  |  |  |  |  |
|  |  |  | 1 | Final elongation | 72 | 02:00 |
| *(Quantabio, Beverly, MA, USA) | | | | | | |

### Archaea

Supplement T 9: PCR settings and composition of reaction mix used for amplification of archaeal 16S rRNA gene sequences.

| **Reagent** | **Volume**  **[µl]** |  | **Cycles** | **Step** | **Temperature**  **[°C]** | **Time**  **[min]** |
| --- | --- | --- | --- | --- | --- | --- |
| 5x Phusion GC buffer* | 10 |  | 1 | Initial denaturation | 98 | 01:00 |
| DEPC H_2_O | 30 |  |  |  |  |  |
| Primer 340f (20 pmol) | 1 |  | 30 | Denaturation | 98 | 00:45 |
| Primer 806r(20 pmol) | 1 |  |  | Primer annealing | 63 (-1 per cycle) | 00:45 |
| MgCl_2_* | 1 |  |  | Elongation | 72 | 00:30 |
| DMSO* | 2.5 |  | 25 | Denaturation | 98 | 00:45 |
| BSA (10 mg/ml) | 1 |  |  | Primer annealing | 51 | 00:45 |
| dNTPs (10 mM) | 1 |  |  | Elongation | 72 | 00:30 |
| Phusion Polymerase | 0.5 |  |  |  |  |  |
| Template | 2 |  | 1 | Final elongation | 72 | 05:00 |
| Sum | 50 |  |  | Hold | 4 | until end |
| *supplied with Phusion High Fidelity DNA Polymerase (New England BioLabs) | | | | |  |  |

### Bacteria

Supplement T 10: PCR settings and composition of reaction mix used for amplification of bacterial 16S rRNA gene sequences.

| **Reagent** | **Volume**  **[µl]** |  | **Cycles** | **Step** | **Temperature**  **[°C]** | **Time**  **[min]** |
| --- | --- | --- | --- | --- | --- | --- |
| 5x Phusion GC buffer* | 10 |  | 1 | Initial denaturation | 98 | 01:00 |
| DEPC H_2_O | 30.8 |  |  |  |  |  |
| Primer V3f (20 pmol) | 1 |  | 30 | Denaturation | 98 | 00:45 |
| Primer V4r(20 pmol) | 1 |  |  | Primer annealing | 60 | 00:45 |
| MgCl_2_* | 0.2 |  |  | Elongation | 72 | 00:30 |
| DMSO* | 2.5 |  |  |  |  |  |
| BSA (10 mg/ml) | 1 |  | 1 | Final elongation | 72 | 05:00 |
| dNTPs (10 mM) | 1 |  |  |  |  |  |
| Phusion Polymerase | 0.5 |  |  |  |  |  |
| Template | 2 |  |  |  |  |  |
| Sum | 50 |  |  | Hold | 4 | until end |
| *supplied with Phusion High Fidelity DNA Polymerase (New England BioLabs) | | | | |  |  |

References

1. Weinbauer, M. G., Fritz, I., Wenderoth, D. F. & Höfle, M. G. Simultaneous extraction from bacterioplankton of total RNA and DNA suitable for quantitative structure and function analyses. *Appl. Environ. Microbiol.* 68, 1082–1087; 10.1128/aem.68.3.1082-1087.2002 (2002).
